# The arabidopsis cyclic nucleotide interactome

**DOI:** 10.1186/s12964-016-0133-2

**Published:** 2016-05-11

**Authors:** Lara Donaldson, Stuart Meier, Christoph Gehring

**Affiliations:** Biological and Environmental Sciences and Engineering Division, King Abdullah University of Science and Technology, Thuwal, 23955-6900 Saudi Arabia; Department of Molecular and Cell Biology, University of Cape Town, Private Bag × 3, Rondebosch, 7701 South Africa

**Keywords:** cAMP, cGMP, Cross-talk, Cyclic nucleotide, Defence response, H_2_O_2_, Hypersensitive response, Nitric oxide, Reactive oxygen species, Second messenger

## Abstract

**Background:**

Cyclic nucleotides have been shown to play important signaling roles in many physiological processes in plants including photosynthesis and defence. Despite this, little is known about cyclic nucleotide-dependent signaling mechanisms in plants since the downstream target proteins remain unknown. This is largely due to the fact that bioinformatics searches fail to identify plant homologs of protein kinases and phosphodiesterases that are the main targets of cyclic nucleotides in animals.

**Methods:**

An affinity purification technique was used to identify cyclic nucleotide binding proteins in *Arabidopsis thaliana*. The identified proteins were subjected to a computational analysis that included a sequence, transcriptional co-expression and functional annotation analysis in order to assess their potential role in plant cyclic nucleotide signaling.

**Results:**

A total of twelve cyclic nucleotide binding proteins were identified experimentally including key enzymes in the Calvin cycle and photorespiration pathway. Importantly, eight of the twelve proteins were shown to contain putative cyclic nucleotide binding domains. Moreover, the identified proteins are post-translationally modified by nitric oxide, transcriptionally co-expressed and annotated to function in hydrogen peroxide signaling and the defence response. The activity of one of these proteins, GLYGOLATE OXIDASE 1, a photorespiratory enzyme that produces hydrogen peroxide in response to *Pseudomonas*, was shown to be repressed by a combination of cGMP and nitric oxide treatment.

**Conclusions:**

We propose that the identified proteins function together as points of cross-talk between cyclic nucleotide, nitric oxide and reactive oxygen species signaling during the defence response.

**Electronic supplementary material:**

The online version of this article (doi:10.1186/s12964-016-0133-2) contains supplementary material, which is available to authorized users.

## Background

The sessile nature of plants demands that they are able to detect and rapidly adapt to changes in their environment. Second messengers are critical to this adaptation as they relay extracellular environmental and developmental signals into intracellular information that is decoded into appropriate physiological responses. A suite of small, transient molecules function as plant second messengers including calcium, cyclic nucleotides (CNs), phospholipids, cyclic ADP ribose, reactive oxygen species (ROS), nitric oxide (NO) and changes in cytosolic pH [1–3].

The CNs, adenosine 3′, 5′-cyclic monophosphate (cAMP) and guanosine 3′, 5′-cyclic monophosphate (cGMP) are well characterized and conserved second messengers that have important functional roles in prokaryotes and eukaryotes [4]. In animals, CNs are synthesised in response to extracellular signals that activate intracellular nucleotidyl cyclases (NCs) which catalyse the synthesis of cAMP and cGMP from their respective nucleotide triphosphate substrates, adenosine triphosphate (ATP) and guanosine triphosphate (GTP). These CNs bind to and activate CN binding proteins (CNBPs) including cAMP- and cGMP-dependent protein kinases (PKA and PKG) which are considered to be the main effectors of CN signaling in animals [5]. In addition, CNs bind to CN-gated and hyperpolarization-activated CN-modulated channels (CNGCs and HCNs) which have specialized roles in sensory perception and control of rhythmicity, respectively [6]. Finally, intracellular CN concentrations are tightly regulated by phosphodiesterases (PDEs) that bind and metabolize cAMP and cGMP to inactive nucleotide monophosphates [4].

Sequence and structural analysis of bacterial and animal CNBPs indicate that CNs bind to conserved CN binding domains (CNBDs). Although different types of CNBPs have been discovered across kingdoms, only two evolutionary distinct CNBDs have been characterized: 1) the CNB domain, present in PKA, PKG, CNGCs, HCNs and the *Escherichia coli* transcription factor, catabolite activator protein (CAP) and 2) the GAF domain, so called because it is found in cGMP binding PDEs, *Anabaena* adenylyl cyclase and *E. coli* FhlA [7].

For decades the presence of CNs in plants was debated [4] but it was not until mass spectrometry (MS) methods were employed that the existence of CNs in plants was proven unequivocally [8]. Since then CNs have been implicated in phytochrome signaling during chloroplast development [9], stomatal movements [10, 11] and responses to: plant hormones [12, 13], ozone [14], NO [12, 15, 16] and abiotic and biotic stresses [17, 18]. Despite this, CN signal transduction in plants is poorly understood because the upstream and downstream components of the pathway remain elusive.

Recently, progress has been made toward the discovery of upstream NCs in plants. Alignment of the catalytic domains of NCs across kingdoms has identified conserved, functionally important amino acids that have been used to construct rational search motifs to query plant genomes and identify candidate NCs [19–21]. To date six putative plant NCs, identified in this way, have been recombinantly expressed and shown to produce CNs in vitro [19, 22–26]. Additionally, overexpression of one of the NC candidates, the phytosulfokine receptor, in protoplasts results in a 20 fold increase in cGMP levels demonstrating that this receptor has NC activity in vivo [24].

Less is known about downstream CN signaling mechanisms in plants. While CN binding activity and CN-modulated protein activity has been demonstrated in oat and morning glory, the corresponding CNBPs have not been identified [27, 28]. Bioinformatics searches for CNBDs in plants, have failed to identify homologs of animal PKA, PKG or PDEs [7]. Plants do however possess an expanded family of CNGCs which suggests that these are the main targets of CN signaling in plants [29]. Other plant proteins that harbour the canonical CNB signature include a family of shaker-type K^+^ channels, acetyl CoA thioesterases, a protein phosphatase 2C (PP2C) and the Na^+^/H^+^ antiporter, SALT OVERLY SENSITIVE 1 (SOS1) [7]. There is experimental evidence to support CN-mediated regulation of CNGCs and K^+^ channels [30, 31], however the function of the CNB site in acetyl CoA thioesterases, PP2C and SOS1 has not yet been explored. On the other hand, GAF domains have been identified in plant phytochromes and ethylene receptors [7]. The fact that CNs play a role in phytochrome signaling suggests these GAF domains are functional [9]. Similarly, ethylene receptors resemble bacterial histidine kinases that contain functional GAF domains [32] and there is some evidence that cGMP plays a role in ethylene signaling [14]. While CNB and GAF domains have been identified in a number of plant proteins, there is little direct evidence that CNs bind and regulate the activity of these proteins.

Plant CNGCs have been studied extensively and shown to facilitate CN activated Na^+^, K^+^ and Ca^2+^ currents [33–38]. Furthermore, *cngc* mutants have revealed functional roles for CNGCs in plant defence against pathogens and particularly the hypersensitive response [39–42], leaf senescence [43], floral transition [44, 45], germination [46], salt tolerance [47, 48], heavy metal tolerance [49], thermotolerance [37, 50], starch accumulation and growth [51, 52], gravitropism [53], polarized tip growth of pollen [54], pollen tube guidance [55], stress tolerance in pollen reproductive development [56] and male reproductive fertility [57]. Additionally, CNGCs have been localized to guard cells suggesting that they play a role in stomatal movements [38]. While CNGCs certainly function in a wide array of physiological processes in plants, the question of whether these are the only effectors of plant CN signalling remains to be answered.

Recent studies have demonstrated that exogenous CN treatment induces changes in the plant transcriptome [58, 59] and phosphoproteome [60] supporting that CNs modulate downstream targets in plants, similar to prokaryotes and other eukaryotes. However, the apparent lack of plant CN-dependent protein kinases and transcription factors suggests that the mechanism by which CNs mediate their effects in plants differs from that in prokaryotes and other eukaryotes. Clearly, the identification of downstream CNBPs is essential to advance our understanding of CN signaling pathways in plants. In the past decade, experimental strategies using synthetic CNs to affinity purify CNBPs have been developed to gain insight into CN signaling in animals. These strategies have been successfully used to isolate known CNBPs (PKA, PKG, PDE, CNGC, HCN and CAP) and identify novel PKA-associated scaffold proteins [61, 62]. Here, we have adapted and applied this methodology to identify CNBPs in *Arabidopsis thaliana*. Twelve CNBPs have been successfully identified and their potential role in plant CN signaling investigated.

## Methods

### Plant material

Arabidopsis Col-0 was grown in a peat/vermiculite mixture at 22 °C and 55 % humidity under a 16 h light (100 μM photons m^−2^ s^−1^) 8 h dark cycle. After four weeks, leaf tissue was harvested. Arabidopsis Col-0 callus cell cultures were grown shaking at 23 °C under continuous light in Gamborg’s B5 media with vitamins, 2 % (*w*/*v*) sucrose, 0.05 % (*w*/*v*) 4-morpholineethanesulfonic acid, 0.5 mg l^−1^ 2,4-dichlorophenoxyacetic acid, 50 μg l^−1^ kinetin at pH 5.7. Cells were sub-cultured weekly, grown for nine days then collected by filtration.

### Chemicals

Synthetic CN baits: 2-(6-aminohexylamino) cAMP agarose (2-AHA-cAMP-agarose); 8-(2-aminoethylamino) cAMP agarose (8-AEA-cAMP-agarose); 2-(6-[Biotinyl] aminohexylamino) cAMP (2-[Biotin]-AHA-cAMP); N2-(6-aminohexyl) cGMP agarose (2-AH-cGMP-agarose); 8-(2-aminoethylthio) cGMP agarose (8-AET-cGMP-agarose); N2-(6-[Biotinyl] aminohexyl) cGMP (2-[Biotin]-AH-cGMP) and the ethanolamine agarose (EtOH-NH-agarose) negative control were purchased from BioLog Life Science Institute (Bremen, Germany). Dynabeads® MyOne™ Streptavidin C1 and Dynabeads® Co-Immunoprecipitation kit were purchased from Invitrogen/Dynal (Oslo, Norway). THE™ cAMP and THE^TM^ cGMP antibodies were purchased from GenScript (Piscataway, NJ).

### Affinity purification overview

Leaf and callus proteins were incubated with four different baits for either cAMP or cGMP. The cAMP baits were: 2-AHA-cAMP-agarose; 8-AEA-cAMP-agarose; 2-[Biotin]-AHA-cAMP and cAMP antibodies while the cGMP baits were: 2-AH-cGMP-agarose; 8-AET-cGMP-agarose; 2-[Biotin]-AH-cGMP and cGMP antibodies (Additional file 1). The synthetic CN baits differed in their linkers (hexyl or ethyl), linkage positions to the nucleotide moiety (2 or 8) and scaffolds (biotin or agarose) and were used to eliminate non-specific binding associated with using a single bait. The antibody baits pulled down proteins bound to endogenous CNs and were used to confirm findings with the synthetic CNs.

### Protein extraction

Approximately 5 g leaf or callus tissue was ground to a fine powder in liquid nitrogen and dissolved in 10 ml assay buffer: 50 mM Tris–HCl pH 7.4, 0.25 M sucrose, 1 mM ethylenediaminetetraacetic acid, 0.1 mM MgSO_4_.7H_2_O, 10 mM KCl, 5 mM ascorbic acid, 1 mM phenylmethanesulfonyl fluoride, 1 × protease inhibitor cocktail (Sigma P9599). Insoluble 0.5 % (*w*/*v*) poly (vinylpolypyrrolidone) was added to remove polyphenols. The protein extracts were centrifuged at 12 000 × *g* for 20 min at 4 °C to obtain a clarified supernatant. The protein concentration of the supernatant was determined, as described previously [63] to be approximately 1 mg ml^−1^. The leaf or callus protein extracts were divided into nine 1 ml aliquots and affinity purified using the four different cAMP baits, four cGMP baits and negative control.

### Affinity purification

#### Agarose baits

In pull-down experiments with agarose baits, 200 μl beads were used, corresponding to approximately 1 nmol cAMP or cGMP. The agarose beads were equilibrated with 1 ml assay buffer in an Eppendorf tube for 2 h at 4 °C with gentle agitation on a rotator at 40 rpm. The beads were collected by centrifugation at 100 × *g* for 30 s and the assay buffer removed. Approximately 1 mg leaf or callus proteins (1 ml protein extract) were incubated with the pre-equilibrated agarose beads for 4 h at 4 °C, with gentle agitation. The protein-bound beads were collected by centrifugation and affinity purified proteins processed as described below.

#### Biotin baits

In pull-down experiments with biotin baits, 1 mg streptadvin-linked dynabeads (capable of binding 2.5 nmol biotin) were used. These were equilibrated with 1 ml assay buffer in an Eppendorf tube for 2 h at 4 °C with gentle agitation on a rotator at 40 rpm, then collected with a Dynamag magnet and the assay buffer removed. Concurrently, approximately 1 mg leaf or callus proteins were pre-incubated with 1 nmol 2-[Biotin]-AHA-cAMP or 2-[Biotin]-AH-cGMP for 1 h at 4 °C with gentle agitation. The biotin-bound proteins were then incubated with the pre-equilibrated streptavidin-linked dynabeads for 4 h at 4 °C, with gentle agitation. The protein-bound dynabeads were collected with a Dynamag and affinity purified proteins processed as described below.

#### Antibody baits

Antibody-coupled dynabeads were generated by covalently coupling 50 μg cAMP or cGMP antibody to 8 mg Dynabeads® M-270 Epoxy using the Dynabeads® Co-Immunoprecipitation kit, according to the manufacturer’s instructions. In each immunoaffinity purification experiment 4 mg antibody-coupled dynabeads were used. The antibody-coupled dynabeads were equilibrated with 1 ml assay buffer for 2 h at 4 °C with gentle agitation on a rotator at 40 rpm then collected with a Dynamag and the assay buffer removed. Approximately 1 mg leaf or callus proteins were incubated with the pre-equilibrated antibody-coupled dynabeads for 4 h at 4 °C, with gentle agitation. The protein-bound dynabeads were collected with a Dynamag, and affinity purified proteins processed as below.

### Affinity purified protein processing

The protein-bound agarose or dynabeads were washed six times with 1 ml assay buffer then subjected to a sequential elution series of increasing stringency and the elution and bead fractions collected, as described previously [63]. The protein concentration of the elution fractions was low, so proteins were precipitated, as described previously [63]. The elution and bead fractions were separated by sodium dodecyl sulphate-polyacrylamide gel electrophoresis and fractionated proteins processed by in-gel tryptic digest, as described previously [63].

### Mass spectrometry

Dried peptides were reconstituted in 12 μl 0.1 % (*v*/*v*) formic acid (FA), 5 % (*v*/*v*) acetonitrile (ACN) and analysed using a Q-TRAP 5500 mass spectrometer (Applied Biosystems, Foster City, CA) connected to a Proxeon nano-liquid chromatography system. For each sample, 5 μl was loaded onto a reverse phase C18 trap column, washed, then eluted at 500 nl min^−1^ using a 3 μM 200°A Magic C18AQ online analytical column connected to a captive-spray source (Michrom, Auburn, CA). Peptides were eluted in a linear gradient of 5–40 % ACN, 0.1 % FA for 25 min; a linear gradient of 40–80 % ACN, 0.1 % FA for 5 min then an isocratic gradient of 80 % ACN, 0.1 % FA for 15 min. Eluted peptides were analysed online by electrospray ionization-MS using Analyst (version 1.5.2) to select MS scans of m/z 300–1000. Each MS scan went through four rounds of MS/MS with dynamic exclusion. The MS/MS data were compared to the Arabidopsis_TAIR10 protein database using Mascot (version 2.3) and results compiled with Scaffold (version 3.6). Positive matches had a peptide identification probability of 95 % and corresponding proteins were represented by at least two peptides with a protein identification probability of 99 %.

### Alignment of candidate proteins with cyclic nucleotide binding domains

Representative CNB domains were downloaded from Interpro (IPR000595) for sequence analysis including *Homo sapiens*: PKAIα [EMBL:P10644]; PKGI [EMBL:Q13976]; HCN1 [EMBL:O60741]; CNGCβ1 [EMBL:P29973]; CNGCα1 [EMBL:Q14028]; exchange protein activated by cAMP (EPAC) 1 [EMBL:O95398] and EPAC2 [EMBL:Q8WZA2] and *E. coli*: CAP [EMBL:P0ACJ8]. GAF domains were retrieved by BLAST searches including *H. sapiens*: PDE2 [EMBL:O00408] and PDE5 [EMBL:O76074]; *Anabaena* sp. PCC 7120 adenylyl cyclases: CYAB [EMBL:P94181] and CYAC [EMBL:P94183] and *E. coli*: FhlA [EMBL:P19323]. Alignments were performed with the Molecular Evolutionary Genetic Analysis (MEGA) 5 program [64].

### Computational analysis of cyclic nucleotide binding proteins

#### Transcriptional co-expression analysis

A transcriptional co-expression analysis was conducted using the Expression Angler tool in Botany Array Resource [65] to determine the level of co-expression shared by each of the candidate CNBPs. The analysis was performed against the NASCArrays 392 microarray dataset, using each of the 12 candidate CNBPs as the driver gene and extracting correlated genes with *r* values between 0.7–1.0. An expression correlation matrix was constructed using the correlated gene list for each CNBP driver gene and extracting *r*-values for the other candidate CNBPs (Table 3).

#### Differential expression analysis

Genevestigator [66] was used to identify experimental conditions that induced the differential expression of the ten expression correlated CNBP candidates from Table 3. Normalised microarray experiments were downloaded from Gene Expression Omnibus [67]: light/dark (GSE9728), *Pseudomonas syringae* pv *maculicola* (*Psm*)/mock (GSE18978), *Pseudomonas syringae* pv *tomato* (*Pst*) DC3000/mock and *Pst* DC3000 *hrpA*/mock (GSE5520). The nitrate starved/replete experiment was downloaded from [68]. Data was processed in excel to generate log_2_ fold change ratios and heat maps were constructed with the MultiExperiment Viewer tool in TMev [69].

### Glycolate oxidase assay

Leaves of four week old Arabidopsis were pressure infiltrated with either 10 mM MgCl_2_ (control) or 10^6^ colony forming units (cfu) ml^−1^*Pst* DC3000 or *Pst* DC3000 *AvrRpm1*. For cGMP and NO treatments, 50 μM 8-Br-cGMP and/or 50 μM diethylamine NONOate were co-infiltrated with MgCl_2_ or *Pst*. GOX activity was measured in *Pst* infected and control plants. Three leaves from each plant were collected 24 h post infection since GOX activity has been shown to contribute to *Pst*-induced H_2_O_2_ production within this time frame. Leaves were ground to a fine powder in liquid nitrogen then added to 500 μl protein extraction buffer and processed as described previously [70]. Briefly, GOX activity in the protein extract catalyses the conversion of the sodium glycolate substrate to glyoxylate, releasing H_2_O_2_. The H_2_O_2_ reacts with O-dianisidine in the presence of horseradish peroxidase to produce the coloured O-dianisidine radical which can be quantified spectrophotometrically at 440 nm [71].

## Results

### Identification of candidate cyclic nucleotide binding proteins

Arabidopsis leaf and callus protein extracts were incubated with four different baits for cAMP or cGMP and the interacting proteins were purified as described previously [63]. A range of baits was used to ensure that specific interactions between proteins and CNs were identified (see Methods) and a sequential elution series was used to displace low affinity binding proteins [61]. A total of 119 proteins were identified from leaf and callus protein extracts, after subtracting proteins that were pulled down with the negative control bait (Additional file 2). The list was filtered to identify the best candidate CNBPs. Briefly, only proteins that were identified with more than one type of bait and that survived the stringent elution process were retained, producing a final list of 13 candidate CNBPs (Table 1; corresponding peptides in Additional file 3). The candidate CNBPs bound tightly to the baits and were not displaced during the sequential elution process. None of the candidate CNBPs bound selectively to the cAMP or cGMP baits. Of the 13 proteins identified, 11 were specific to leaf, one was found in both leaf and callus and one was unique to callus. Notably, 12 of the 13 CNBP candidates were purified with CN-specific antibodies supporting that these proteins bind endogenous CNs. The 13 candidate CNBPs identified were subsequently analysed for potential roles in CN signaling.

**Table 1:** Arabidopsis cyclic nucleotide binding protein candidates

		LEAF cAMP				LEAF cGMP				CALLUS cAMP				CALLUS cGMP			
Accession	Description	2-AHA-cAMP-agarose	8-AEA-cAMP-agarose	2-[Biotin]-AHA-cAMP	cAMP antibody	2-AH-cGMP-agarose	8-AET-cGMP-agarose	2-[Biotin]-AH-cGMP	cGMP antibody	2-AHA-cAMP-agarose	8-AEA-cAMP-agarose	2-[Biotin]-AHA-cAMP	cAMP antibody	2-AH-cGMP-agarose	8-AET-cGMP-agarose	2-[Biotin]-AH-cGMP	cGMP antibody
AT3G13920	eukaryotic translation initiation factor 4A1			B		B			B	B	B	B	B	B	B	B	B
AT3G12780	phosphoglycerate kinase 1	B	B	B	B	B	B	B	B								
AT1G42970	glyceraldehyde-3-phosphate dehydrogenase B subunit	B	B	B	2, 3, B	B	B	B	B								
AT4G20360	RAB GTPase homolog E1B	B	B	B	2, 3, B	B	B	B	B								
AT3G60750	Transketolase		B	B	B	B	B	B	B								
AT5G50920	CLPC homologue 1	B	B	B	B	B	B	B									
AT4G37930	serine transhydroxymethyltransferase 1	2, B	B	1, B	B	B	B	2, 3, B									
AT3G14420	Glycolate oxidase 1		B		B			B	B								
AT4G09650	ATP synthase delta subunit			B		B		B	B								
AT1G09340	chloroplast stem-loop binding protein of 41 kDA			1	B	B		2, B									
AT1G56330 AT3G62560 AT4G02080	secretion-associated RAS 1B									B					B		B
AT4G27440	protochlorophyllide oxidoreductase B		B		B	B											
AT3G01500	carbonic anhydrase 1			B		B											

The fraction in which the protein was identified is indicated with numbers 1–6 being the elution fractions (1 denotes the first and least stringent elution and 6 the final and most stringent elution) while B indicates that the protein was found in the bead fraction. For the cAMP baits the elutions were 1) 100 mM GDP; 2) 100 mM AMP; 3) 10 mM cGMP; 4) 100 mM cGMP; 5) 10 mM cAMP and 6) 100 mM cAMP. For the cGMP baits the elutions were 1) 100 mM ADP; 2) 100 mM GMP; 3) 10 mM cAMP; 4) 100 mM cAMP; 5) 10 mM cGMP and 6) 100 mM cGMP

### Candidate CNBPs have putative cyclic nucleotide binding domains

The candidate CNBPs were queried against the UniProt and Interpro databases [72, 73] to determine whether they contain binding domains or binding sites for cyclic or similar nucleotides. During this analysis it was noted that peptides mapping to SECRETION ASSOCIATED RAS 1B do not retrieve a single protein but rather a family of similar proteins. As it is impossible to determine which of these bound the bait, all were excluded from further analysis. The search revealed that none of the candidate CNBPs are annotated to bind CNs. However, several candidate CNBPs are annotated to bind adenine and guanine containing nucleotides (Table 2). The ATP binding proteins include EUKARYOTIC TRANSLATION INITIATION FACTOR 4A1 (EIF4A1), PHOSPHOGLYCERATE KINASE 1 (PGK1) and CASEINOLYTIC PROTEASE C HOMOLOG 1 (CLPC1). The NAD/NADP binding proteins include GLYCERALDEHYDE-3-PHOSPHATE DEHYDROGENASE B SUBUNIT (GAPB), CHLOROPLAST STEM-LOOP BINDING PROTEIN OF 41 kDa (CSP41B) and PROTOCHLOROPHYLLIDE OXIDOREDUCTASE B (PORB). Finally, RAS-RELATED IN BRAIN GTPase HOMOLOG E1b (RABE1b) binds GTP (Table 2). The remaining candidate CNBPs have no known affinity for other nucleotides including GLYCOLATE OXIDASE (GOX1), TRANSKETOLASE (TKL), SERINE HYDROXYMETHYLTRANSFERASE 1 (SHMT1), ATP SYNTHASE DELTA-SUBUNIT (ATPD) and CARBONIC ANHYDRASE 1 (CA1).

**Table 2:** Binding properties of the cyclic nucleotide binding protein candidates

Accession	Description	Nucleotide binding	Nucleotide binding domain	Nucleotide binding site	Alignment with CNBD	Putative CNBD	NO PTM	Site of NO PTM
AT3G13920	eukaryotic translation initiation factor 4A1	ATP	58–255	83–90	GAF	174–399	S-nitrosylation	
AT3G12780	phosphoglycerate kinase 1	ATP		432–435	CNB GAF	87–286 171–335	S-nitrosylation S-nitrosylation^HR^	
AT1G42970	glyceraldehyde-3-phosphate dehydrogenase B subunit	NAD (P)	80–243	91–92	CNB GAF	173–323 83–288	S-nitrosylation Y-nitration	
AT4G20360	RAB GTPase homolog E1B	GTP	77–278	86–93 148–152 203–206			S-nitrosylation S-nitrosylation^HR^ Y-nitration	
AT3G60750	Transketolase				CNB GAF	213–408 40–232	S-nitrosylation Y-nitration	Y337
AT5G50920	CLPC homologue 1	ATP	294–434 637–818	302–309 645–652				
AT4G37930	serine transhydroxymethyltransferase 1				CNB GAF	369–492 262–458	Y-nitration	
AT3G14420	Glycolate oxidase 1	FMN	13–355	285–309	GAF	93–276	Y-nitration	
AT4G09650	ATP synthase delta-subunit							
AT1G09340	chloroplast stem-loop binding protein of 41 kDA	NAD (P)	54–235				S-nitrosylation S-nitrosylation^HR^ Y-nitration	
AT4G27440	protochlorophyllide oxidoreductase B	NAD (P)	84–234 270–366		CNB	77–279	Y-nitration	
AT3G01500	carbonic anhydrase 1				GAF	141–345	S-nitrosylation S-nitrosylation^HR^ Y-nitration	C280

**Fig. 1:**
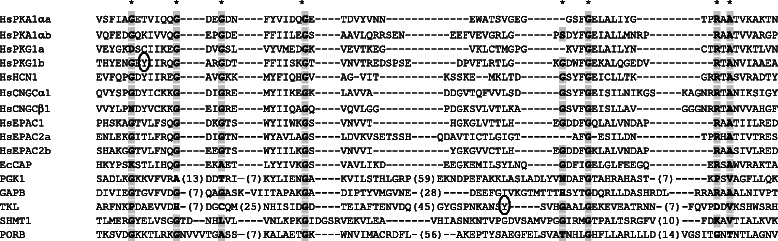
Alignment of candidate cyclic nucleotide binding proteins with known CNB domains. Representative CNB domains were obtained from Interpro (IPR000595) including those from human (Hs) protein kinases PKA1α, PKG1; channels HCN1, CNGCα1, CNGCβ1; and guanine nucleotide exchange factors EPAC1, EPAC2 and *E. coli* (Ec) transcription factor CAP. Where the protein has tandem CNB domains these are indicated by the letters “a” and “b”. The number in parenthesis indicates the number of amino acids that have been omitted. The * indicates conserved amino acids across species. Tyrosine residues that have been circled are sites of Y-nitration. The Arabidopsis CNBP candidates PGK1, GAPB, TKL, SHMT1 and PORB align with the CNB domain. The site of Y-nitration in the CNBP candidate, TKL is found within the CNB domain although it is distant from the Y-nitration site in human PKG

**Fig. 2:**
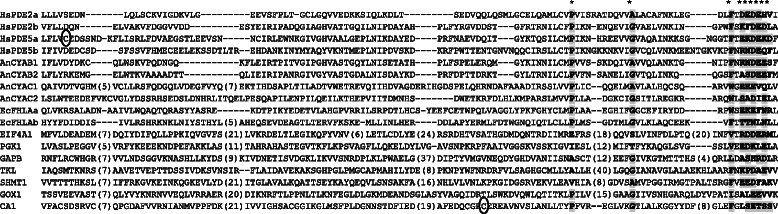
Alignment of candidate cyclic nucleotide binding proteins with known GAF domains. Representative GAF domains (IPR003018) include those from human (Hs) phosphodiesterases PDE2, PDE5; *Anabaena* sp. PCC 7120 (An) adenylyl cyclases CYAB1, CYAB2, CYAC1, CYAC2; and *E. coli* (Ec) FhlA. Where the protein has tandem GAF domains these are indicated by the letters “a” and “b”. The number in parenthesis indicates the number of amino acids that have been omitted. The * indicates conserved amino acids across species. The cysteine residues that have been circled are sites of S-nitrosylation. The Arabidopsis CNBP candidates EIF4A1, PGK1, GAPB, TKL, SHMT1, GOX1 and CA1 align with the GAF domain. The site of S-nitrosylation in the CNBP candidate CA1 is found within the GAF domain although it is distant from the S-nitrosylation site in human PDE5.

Annotated nucleotide binding domains and nucleotide binding sites of the candidate CNBPs were extracted from the Uniprot and Interpro databases. The alignment of the candidate CNBPs with known CNBDs was extrapolated from Figs. 1 and 2. Evidence of NO-induced PTM and the PTM site was obtained from the literature [79–82]

To investigate whether the candidate CNBPs contain potential CNBDs, their sequences were aligned with known CNBDs. To achieve this, CNB domains were extracted from representative organisms across kingdoms including human, mouse, chicken, frog, fish, fly, worm, slime mould, protozoa, yeast, bacteria, cyanobacteria, algae, moss, Arabidopsis and rice. Since the GAF domain is less constrained than the CNB domain, only published GAF domains from human, yeast, bacteria, cyanobacteria, Arabidopsis and sorghum [74–78] were considered. More than 200 CNB sequences and 50 GAF sequences were aligned with the identified Arabidopsis candidate CNBPs (Additional files 3 and 4). The alignment results were simplified to show only the candidate CNBPs that did align with select CNB and GAF domains from well-known CNBPs (Figs. 1 and 2). Eight of the candidate CNBPs have sequences that resemble known CNBDs (CNBD-like sequences) with several candidates aligning with both CNB and GAF domains (Table 2). Specifically, CNB-like sequences were identified in PGK1, GAPB, TKL, SHMT1 and PORB while GAF-like sequences were identified in EIF4A1, PGK1, GAPB, TKL, SHMT1, GOX1 and CA1. Only RABE1b, CLPC1, ATPD and CSP41B did not align with either CNBD. Thus, eight of the 12 candidate CNBPs contain CNBD-like sequences, validating the experimental approach.

### Candidate CNBPs are modified by nitric oxide

An investigation of The Arabidopsis Information Resource (TAIR) database revealed that a number of the candidate CNBPs are annotated to be post-translationally modified (PTM) by NO. Further investigation of the literature revealed that ten of the candidate CNBPs have been experimentally determined to be modified by NO with several being modified by both S-nitrosylation and Y-nitration [79–82] (Table 2). Specifically, EIF4A1, PGK1, GAPB, RABE1b, TKL, CSP41B and CA1 are modified by S-nitrosylation while GAPB, RABE1b, TKL, SHMT1, GOX1, CSP41B, PORB and CA1 are modified by Y-nitration. Of the ten NO-modified candidate CNBPs, seven also contain CNBD-like sequences. For two of the ten NO-modified candidate CNBPs, TKL and CA1, the PTM site has been determined [81, 83]. In TKL, the modified tyrosine (Y337) lies within the putative CNB domain (Table 2) and this tyrosine is conserved in CNB domains of *E. coli* CAP and type II PKAs (Fig. 1, Additional file 4). Similarly, in CA1 the nitrosylated C280 lies within the GAF domain and a nearby cysteine is conserved in all plant and bacterial phytochromes (Additional file 5). This revealed that a number of candidate CNBPs are modified by NO and, for TKL and CA1, the PTM site lies within the putative CNBD supporting that CNs and NO can modify these proteins at the same site and presenting a possible mechanism of cross-talk between these second messengers.

### Six of the candidate CNBPs are Calvin cycle and photorespiratory enzymes

**Fig. 3:**
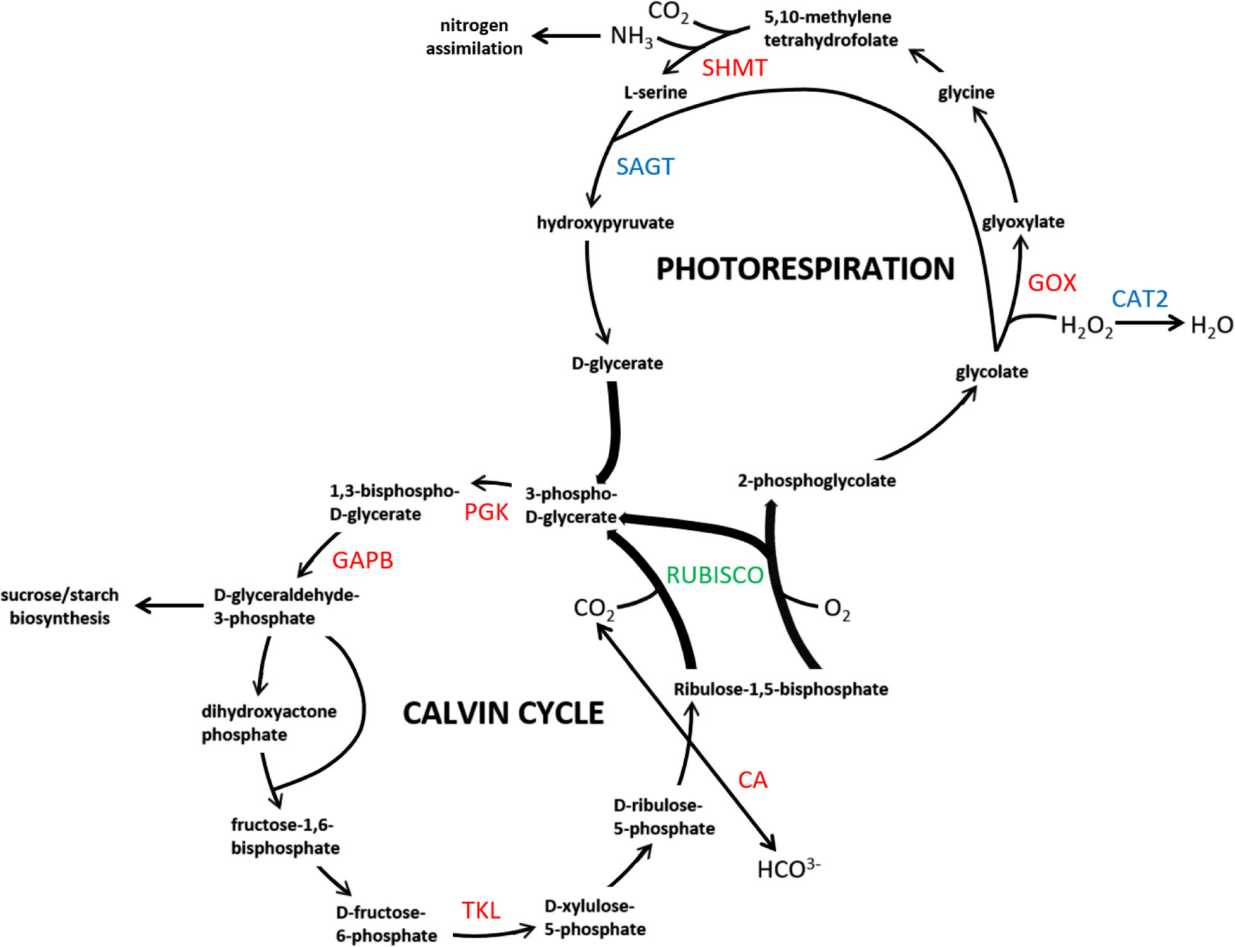
The Calvin cycle and photorespiration pathway. The CNBP candidates are depicted in red font. Additional photorespiratory enzymes that were pulled down during the affinity purification procedure but excluded during the stringent elimination process (Table S2) are shown in blue font, where SAGT is ALANINE: GLYOXYLATE AMINOTRANSFERASE and CAT2 is CATALASE 2. The Calvin cycle and photorespiration pathway are connected by the dual-functioning enzyme RUBISCO shown in green. The CNBP candidate CA1 interconverts soluble HCO^3−^ to gaseous CO_2_ and controls the supply of CO_2_ to RUBISCO and regulates stomatal closure through HCO^3−^ effects on anion channels thereby further affecting the supply of CO_2_ to the plant. The CNBP candidates PGK1, GAPB and TKL are enzymes in the Calvin cycle. PGK1 and GAPB convert Ribulose-1, 5-bisphosphate (RuBP) into the triose phosphate, D-glyceraldehyde-3-phosphate which can feed into sucrose and starch biosynthesis. TKL is involved in the regeneration of RuBP and significantly controls carbon flux through the Calvin cycle. The CNBP candidates GOX1 and SHMT1 are enzymes in the photorespiration pathway. GOX1 catalyses the conversion of glycolate to glyoxylate with the concomitant release of H_2_O_2_ as a by-product. SHMT1 converts two molecules of glycine to serine, CO_2_, NH_3_ and NADH. This CO_2_ can feedback into the Calvin cycle while NH_3_ can feed into nitrogen assimilation

The TAIR descriptions further revealed that a number of the candidate CNBPs function in the Calvin cycle or photorespiration pathway. The Calvin cycle utilizes ATP and NADPH to assimilate CO_2_ into carbon skeletons [84] while photorespiration consumes ATP and NADH to regenerate CO_2_; and is important for limiting photoinhibition, nitrate assimilation and ROS signaling [85]. The positions of the candidate CNBPs in the Calvin cycle and photorespiration pathways are illustrated in Fig. 3.

The Calvin cycle is connected to the photorespiration pathway by RIBULOSE-1, 5-BISPHOSPHATE CARBOXYLASE/OXYGENASE (RUBISCO) that either binds CO_2_ or O_2_ to catalyse the carboxylation or oxygenation of D-ribulose-1, 5-bisphosphate (RuBP) to initiate the Calvin cycle or photorespiration, respectively. The balance between the Calvin cycle and photorespiration is determined, in part, by the supply of CO_2_ to RUBISCO [85]. This is controlled by the candidate CNBP, CA1 which converts HCO^3−^ to CO_2_ at the site of RUBISCO [86]. Thereafter, the initial reductive steps of the Calvin cycle are performed by PKG and GAPB that convert the product of RuBP carboxylation to a triose phosphate that can feed into sucrose and starch biosynthesis. Later in the Calvin cycle, TKL regenerates RuBP and regulation of TKL significantly controls carbon flux through the cycle [84]. Therefore the candidate CNBPs CA1, PKG, GAPB and TKL are key enzymes that regulate the supply, removal and flux of carbon through the Calvin cycle.

Early in the photorespiration pathway, GOX1 catalyses the oxidation of glycolate to glyoxylate with the concomitant release of H_2_O_2_. Later in the pathway SHMT1 converts two molecules of glycine to serine, CO_2_, NH_3_ and NADH. Thus the candidate CNBPs, GOX1 and SHMT1 are key enzymes in the photorespiration pathway that catalyse reactions whose by-products (H_2_O_2_, NH_3_ and CO_2_) are important for ROS signaling, nitrogen assimilation or feedback to the Calvin cycle.

### Candidate CNBPs function in H_2_O_2_ signaling and defence

While the enzymatic activities of the above six candidate CNBPs are known and their functions related, there is little information available for the other six candidate CNBPs. Moreover, the candidate CNBPs with known enzyme activities are involved in primary metabolism so it is unclear under what circumstances their regulation is important. Therefore a transcriptional co-expression, gene ontology (GO) and stimulus-specific expression analysis was performed, to better understand the functions of the candidate CNBPs [87].

The transcriptional co-expression analysis was conducted to determine the level of co-expression shared by each of the candidate CNBPs and identify potential functional relationships between their genes/proteins. It was determined that expression of ten of the 12 candidate CNBPs is highly correlated including the Calvin cycle and photorespiratory genes and the less well functionally characterized ATPD, RABE1b, CLPC1 and CSP41B (Table 3). The high level of co-expression of these four genes with the Calvin cycle and photorespiratory genes suggests that they may have functional roles in these processes. The two candidate CNBPs that were not significantly correlated included EIF4A and PORB.

**Table 3:** Expression correlation matrix of cyclic nucleotide binding protein candidates

EIF4A	PGK1	GAPB	RABE1b	TKL	CLPC1	SHMT1	GOX1	ATPD	CSP41B	PORB	CA1	
1	X	X	X	X	X	X	X	X	X	X	X	EIF4A
	1	0.956	0.876	0.877	0.834	0.893	0.897	0.910	0.958	X	0.947	PGK1
		1	0.900	0.886	0.783	0.851	0.923	0.922	0.973	X	0.940	GAPB
			1	0.813	0.736	0.770	0.856	0.881	0.881	X	0.867	RABE1b
				1	0.77	0.763	0.818	0.826	0.859	X	0.858	TKL
					1	0.784	0.756	0.718	0.785	X	0.751	CLPC1
						1	0.803	0.756	0.817	X	0.839	SHMT1
							1	0.881	0.920	X	0.898	GOX1
								1	0.942	X	0.900	ATPD
									1	X	0.946	CSP41B
										1	X	PORB
											1	CA1

Expression correlation matrix detailing the level of co-expression (expression correlation *r*-value) that the identified CNBP candidates share with each other. X denotes that the shared *r*-value was below 0.7

A GO analysis of the ten expression correlated CNBP candidates (from Table 3), using the TAIR database, revealed that seven are annotated to function in the defence response while eight have GO terms related to H_2_O_2_ signaling (Table 4). Only RABE1b did not have GO terms associated with either defence or H_2_O_2_, however its co-expression with the other expression correlated CNBP candidates suggests that it plays a role in these processes [23, 88]. This analysis supports that the expression correlated CNBP candidates function in H_2_O_2_ signaling and the defence response. These functions could be related as H_2_O_2_ signaling is critical to the defence response, particularly the hypersensitive response (HR) during incompatible interactions with avirulent pathogens [89]. In support of this, six of the expression correlated CNBP candidates have GO annotations for both H_2_O_2_ signaling and the defence response while five have GO terms for “incompatible interaction”; “plant-type HR” or “regulation of plant-type HR”.

**Table 4:** Gene ontology biological process annotations of the expression correlated cyclic nucleotide binding protein candidates

Accession	Description	Defence response	H_2_O_2_ signaling
AT3G12780	phosphoglycerate kinase 1	defence response to bacterium, defence response, incompatible interaction	hydrogen peroxide catabolic process
AT1G42970	glyceraldehyde-3-phosphate dehydrogenase B subunit	defence response to bacterium	hydrogen peroxide catabolic process
AT4G20360	RAB GTPase homolog E1B		
AT3G60750	Transketolase		hydrogen peroxide catabolic process
AT5G50920	CLPC homologue 1		hydrogen peroxide catabolic process
AT4G37930	serine transhydroxymethyl-transferase 1	defence response to bacterium, defence response, incompatible interaction, plant-type hypersensitive response, salicylic acid biosynthetic process	
AT3G14420	Glycolate oxidase 1	defence response to bacterium	hydrogen peroxide biosynthetic process
AT4G09650	ATP synthase delta-subunit	defence response to bacterium, defence response to fungus, defence response, incompatible interaction, detection of biotic stimulus, jasmonic acid mediated signaling pathway, negative regulation of defence response, regulation of plant-type hypersensitive response, regulation of response to biotic stimulus, response to chitin, salicylic acid biosynthetic process, systemic acquired resistance, salicylic acid mediated signaling pathway	regulation of hydrogen peroxide metabolic process
AT1G09340	chloroplast stem-loop binding protein of 41 kDA	defence response to bacterium, defence response to fungus, defence response, incompatible interaction, detection of biotic stimulus, jasmonic acid mediated signaling pathway, negative regulation of defence response, regulation of plant-type hypersensitive response, regulation of response to biotic stimulus, salicylic acid biosynthetic process, systemic acquired resistance, salicylic acid mediated signaling pathway	regulation of hydrogen peroxide metabolic process
AT3G01500	carbonic anhydrase 1	defence response to bacterium, defence response to fungus, defence response to fungus, incompatible interaction, defence response, incompatible interaction, detection of biotic stimulus, jasmonic acid mediated signaling pathway, negative regulation of defence response, regulation of plant-type hypersensitive response, regulation of response to biotic stimulus, response to chitin, salicylic acid biosynthetic process, systemic acquired resistance, salicylic acid mediated signaling pathway	regulation of hydrogen peroxide metabolic process

The GO biological process terms related to the plant defence response against pathogens and H_2_O_2_ signaling were downloaded from TAIR for the ten expression correlated CNBPs extracted from Table 3

**Fig. 4:**
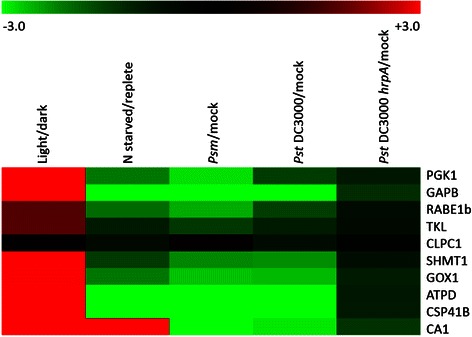
Gene expression profiling of the expression correlated cyclic nucleotide binding protein candidates. Heat map to show that the ten expression correlated CNBP candidates (extracted from Table 3) are co-expressed and differentially expressed in the selected microarray experiments. Experiments chosen were light vs. dark grown seven day old seedlings; seven day old seedlings that were shifted to nitrogen starved vs. replete media for a further 48 h; Col-0 infected with 10^5^ cfu cm^−2^ virulent *Psm* ES4326 for 24 h vs. mock infected; Col-0 infected with 10^8^ cfu cm^−2^ virulent *Pst* DC3000 or 10^8^ cfu cm^−2^ *Pst* DC3000 *hrpA* mutant (lacks the type III protein secretion system that delivers virulence effector proteins into host cells) for 7 h vs. mock infected. The scale bar shows the intensity of the log_2_ transformed fold change values used to generate the heat map

Finally, an *in silico* global expression analysis was performed to identify conditions that induce differential expression of the ten expression correlated CNBP candidates. Genevestigator was used to screen the publically available microarray data and identify experiments of interest. The expression correlated CNBP candidates were found to be induced in response to light and repressed in response to nitrate starvation, like other photorespiratory genes [85]; and repressed in plants infected with *Pseudomonas* (Fig. 4). The stimulus-specific expression profile confirms that the expression correlated CNBP candidates have similar expression profiles and supports that they play a role in photorespiration and the defence response.

### Glycolate oxidase activity is repressed by cGMP and nitric oxide

Since a number of the expression correlated CNBP candidates are modified by NO and annotated to function in H_2_O_2_ signalling and the defence response, it is conceivable that CN binding and NO-mediated PTM could regulate the activity of these proteins to modify H_2_O_2_ signalling in the plant response to pathogens. To test this hypothesis, the activity of GOX1 was examined as it has been shown to produce H_2_O_2_ during the defence response [70].

**Fig. 5:**
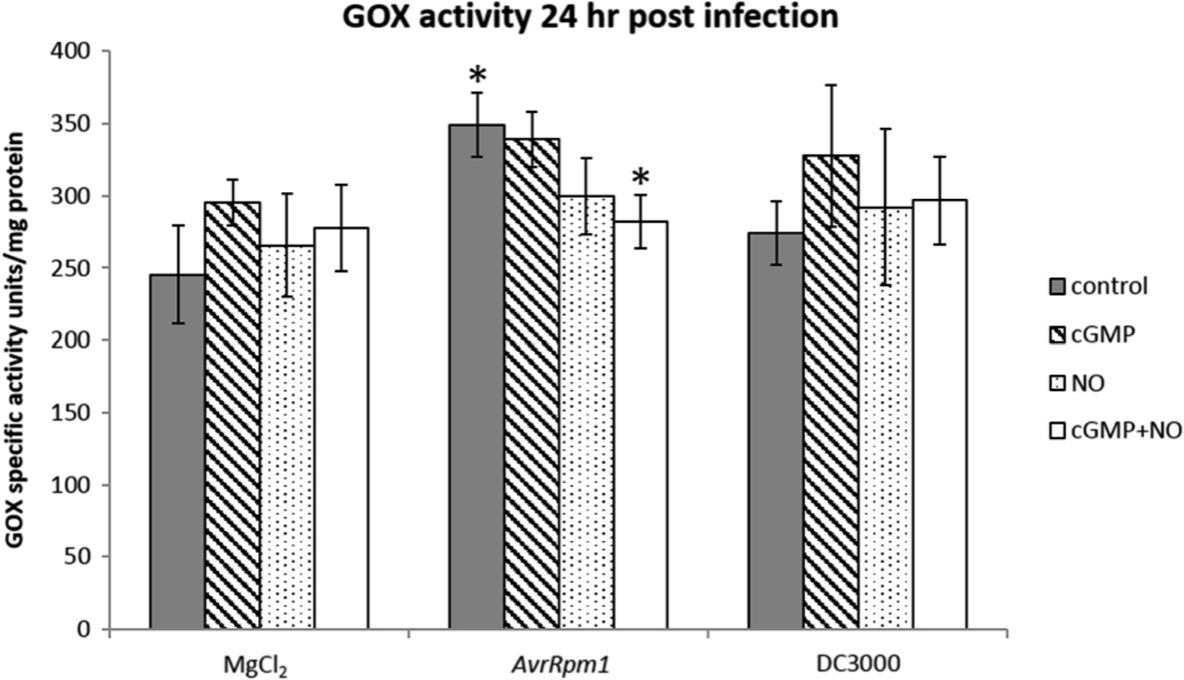
Glycolate oxidase activity in plants inoculated with avirulent and virulent *Pseudomonas*. The leaves of four week old Col-0 plants were pressure inoculated with either 10^6^ cfu ml^−1^ avirulent *Pst* DC3000 *AvrRpm1* or virulent *Pst* DC3000 or 10 mM MgCl_2_ (control). The effect of NO and cGMP during infection was examined by including 50 μM 8-Br cGMP or 50 μM diethylamine NONOate either separately or in combination, in the bacterial suspension at the time of infection. The infected leaves were harvested at 24 h post infection and GOX activity measured. Leaf proteins were extracted and incubated with sodium glycolate, O-dianisidine and horseradish peroxidase. GOX activity catalyses the conversion of the sodium glycolate substrate to glyoxylate, releasing H_2_O_2_. The H_2_O_2_ reacts with O-dianisidine in the presence of horseradish peroxidase to produce the coloured O-dianisidine radical which can be quantified spectrophotometrically at 440 nm. GOX activity was found to be significantly induced (*p* = 0.0276) by *ArvRpm1* relative to the MgCl_2_ control and this activity was significantly suppressed (*p* = 0.0400) by the combined treatment with NO and cGMP. Error bars represent standard error of the mean (*n* = 6) and statistical significance was determined using a student’s t-test with the asterisks denoting significant *p* values < 0.05

The effect of cGMP and NO on GOX activity was measured during the plant response to pathogens. Plants infected for 24 h with avirulent *Pst* DC3000 *AvrRpm1*, but not virulent *Pst* DC3000, were found to have significantly (*p* = 0.0276) increased GOX activity (Fig. 5). When applied alone, neither cGMP nor NO had any effect on *Pst* DC3000 *AvrRpm1*-induced GOX activity. However, in combination, cGMP and NO significantly (*p* = 0.0400) repressed *Pst* DC3000 *AvrRpm1*-induced GOX activity to basal levels. These results indicate that NO and cGMP repress GOX activity, and thus H_2_O_2_ production, during the defence response which is consistent with regulation of GOX1 through cGMP binding and NO-mediated PTM.

## Discussion

In plants, cGMP and cAMP have been shown to play an important role in physiological processes including stomatal closure and the defence response [10, 16]. While a number of NCs have been identified in Arabidopsis and demonstrated to synthesize CNs, little is known about the cytosolic target proteins and thus the downstream mechanisms of cGMP and cAMP signaling. Here, we attempted to elucidate these mechanisms using an affinity pull-down approach to identify CNBPs.

### Experimental protocol validation

Twelve candidate CNBPs were identified from Arabidopsis leaf and callus extracts using an affinity pull-down technique with synthetic cAMP and cGMP baits and CN-specific antibodies (Table 1). Candidate CNBPs displayed high affinity binding but lacked specificity for cAMP or cGMP baits. In animal studies similar observations have been made for the well-known CNBPs, PKA and PKG [61, 90]. Indeed, cAMP and cGMP bind each other’s kinases and PDEs suggesting that extensive cross-talk occurs between cAMP and cGMP signaling pathways [91]. The majority of candidate CNBPs identified were isolated from leaf extracts. This could be attributed to CN signaling being prevalent in leaf tissue where CNs function in chloroplast development, stomatal signaling and stress responses [9, 10, 17, 18]. In contrast, undifferentiated callus may lack functional components of these processes.

There have been three previous attempts to identify CNBPs in plants using synthetic CN baits however each of these studies used only one CN bait and produced limited information. One study failed to identify the interacting proteins [92]. The other two studies identified nucleoside diphosphate kinases which were detected in this study and in animal studies but were discarded as low affinity binding proteins as they were displaced during the sequential elution process [10, 93]. The only other protein identified was GAPB which was also identified here [93].

### Sequence analysis of candidate CNBPs

Sequence analysis revealed that none of the identified Arabidopsis candidate CNBPs contain annotated CNBDs. The majority of Arabidopsis proteins harbouring CNBDs are membrane-associated channels and K^+^ transporters. These are unlikely to be present in our experiments since the protein extraction buffer would most likely isolate soluble proteins and not hydrophobic membrane proteins. Consistent with this, animal membrane associated CNBPs have proved difficult to purify using CN baits [62]. The aim of this study however, was to identify unknown downstream components of Arabidopsis CN signaling pathways, such as CN-dependent protein kinases and PDEs which are soluble proteins. Additionally, since CNs are synthesised in the cytosol, it is reasonable to assume that direct targets of CNs would be found in the soluble protein fraction.

In contrast to the lack of annotated CNBDs, a number of the candidate CNBPs are annotated to bind related nucleotides such as ATP or GTP (Table 2). It is considered unlikely that these candidate CNBPs bind the CN baits with low affinity because of their affinity for similar nucleotides since they were not displaced during the sequential elution process. Furthermore all of the candidate CNBPs, except CA1, were immunoaffinity purified with antibodies which are highly specific to cAMP and cGMP. This is significant as it is possible that proteins bind the synthetic CNs due to excess CN concentrations. However, in the immunoaffinity pull-downs no exogenous CNs were added so the interaction was dependent on the protein binding to endogenous CNs. In animals, PKA and PKG bind ATP through their kinase domains while EPACs bind GTP through guanine nucleotide exchange factor domains. Thus it is not unexpected that CNBPs bind other nucleotides in addition to CNs.

Alignment of the candidate CNBPs with known CNBDs, revealed that eight of the 12 candidate CNBPs contain sequences that are similar to CNB or GAF domains (Figs. 1 and 2). There is no detectable overlap between the abovementioned nucleotide binding sites and the putative CNBDs, supporting that the candidate CNBPs possess distinct CN and nucleotide binding sites (Table 2). These CNB- and GAF-like sequences are not annotated in the protein databases as search motifs employed by these databases are conservative; whereas the CNBD-like sequences identified here allow for single mismatches, insertions or deletions or larger gap regions between stretches of conserved amino acids. Importantly similar CNBD-like sequences have been identified in *Dictyostelium* cGMP binding proteins (GbpA-D), and these have been shown to bind CNs (Additional file 4) [94, 95]. Another possibility is that plants have evolved unique CNBDs that have yet to be annotated. The experimental design here allows for this possibility; however we could not identify any novel CNBDs from the alignments.

### Nitric oxide-mediated post translational modification

In animals, NO is intrinsically linked to cGMP signaling since NO stimulates the soluble guanylyl cyclase (GC) to produce cGMP [96]. It is plausible that a similar NO/cGMP signaling pathway operates in plants since NO has been shown to induce cGMP synthesis in plants [12, 15] and a NO-responsive GC was recently identified in Arabidopsis [26].

The finding that ten of the 12 candidate CNBPs are modified by NO-induced PTMs, S-nitrosylation and Y-nitration, (Table 2) was interesting as it presents a potential mechanism for cross-talk between cGMP and NO signalling pathways. This result is particularly significant when considering that only 120 and 130 Arabidopsis proteins have been demonstrated to be S-nitrosylated and Y-nitrated, respectively under basal conditions and/or during the HR [79–82]. It was not possible to detect either PTM in our experiments; not surprisingly as these PTMs are in low abundance and labile and thus typically studied using the biotin-switch technique. Furthermore, S-nitrosylation and Y-nitration may have been destroyed during the electrophoresis and tryptic digest which included reducing agents [80, 81]. Even using the biotin-switch technique, PTM sites have been difficult to detect. Nevertheless for the candidate CNBPs, TKL and CA1, the PTMs have been mapped and in both cases the PTM site is contained within the putative CNBD (Figs. 1 and 2). Conservation of these cysteine and tyrosine residues across kingdoms is indicative of functional importance (Additional files 4 and 5).

In agreement with our finding that the Arabidopsis candidate CNBPs are subject to NO-mediated PTM, NO has been shown to modify animal CNBPs. There is evidence that Y-nitration of PKA decreases affinity for cAMP binding, although the Y-nitration site remains unknown [97]. Similarly, Y-nitration of PKG at Y247, within the CNB domain, has been shown to decrease PKG activity [98]. This site lies upstream of the Y-nitration site in TKL, however a tyrosine at this position is conserved in HCNs and CNGCs and in the candidate CNBP, SHMT1 that is also subject to Y-nitration (Fig. 1; Additional file 4). Additionally, there is evidence for S-nitrosylation of PDE5A at C181, within the GAF domain, that reduces PDE activity by interfering with cGMP binding [99]. This site is not conserved in other known GAF domains however there is a similar site in GAPB, a CNBP candidate that is subject to S-nitrosylation (Fig. 2; Additional file 5). Therefore, Y-nitration of the CNB domain and S-nitrosylation of the GAF domain may be a general mechanism of NO-mediated regulation of CNBPs and cross-talk between cGMP and NO signaling pathways that is conserved across plant and animal kingdoms.

### Functions of the candidate CNBPs

Six of the identified Arabidopsis candidate CNBPs are key enzymes in the Calvin cycle and photorespiration pathway (Fig. 3). Transcriptional co-expression analysis revealed that the Calvin cycle and photorespiratory genes are significantly co-expressed with the less well functionally characterized CNBPs, ATPD, RABE1b, CLPC1 and CSP41B (Table 3). These lessor known genes may play a role in these pathways since a number of studies have shown that co-expressed genes often function together in common biological processes [100, 101].

Modulation of the activities of these enzymes, through CN binding or NO-mediated PTM, may affect the balance between respiration and photorespiration which would alter H_2_O_2_ levels synthesized during photorespiration [85]. In support of this, eight of the abovementioned ten expression correlated CNBP candidates are annotated to function in H_2_O_2_ signaling (Table 4). Previously it has been shown that the activity of GOX1, the photorespiratory enzyme that produces H_2_O_2_ (Fig. 3), is modified by NO and this has been proposed to regulate H_2_O_2_ levels in response to abiotic stresses [102]. We have shown that GOX1 binds CNs and contains a GAF-like domain, suggesting that CNs could also affect the activity of GOX1 to regulate H_2_O_2_ levels.

In plants cGMP, NO and H_2_O_2_ signaling pathways have well established roles in two processes: stomatal closure and the defence response, particularly the HR during incompatible interactions with avirulent pathogens [2, 16, 103–105]. During ABA-mediated stomatal closure, ABA-induced NO and H_2_O_2_ stimulate cGMP synthesis which leads to increases in cytosolic Ca^2+^, likely through cGMP activation of CNGCs [10]. Subsequently Joudoi and colleagues have shown that reactive nitrogen species (produced when ROS react with NO) react with cGMP to produce 8-Nitro-cGMP and this triggers the increase in cytosolic Ca^2+^ which then acts on SLAC1 anion channels to induce stomatal closure [105]. Two CNGCs, CNGC5 and CNGC6, are likely to be responsible for these cGMP activated Ca^2+^ currents in guard cells [38]. However, the fact that *cngc5cngc6* double mutants are not impaired in ABA-induced stomatal closure suggests that there are other factors involved, consistent with the observation that cGMP is required but not sufficient for ABA-induced stomatal closure [10]. We would suggest that these other factors involved in ABA-induced stomatal closure include the identified CNBP candidates. For example, CA1 could be a downstream target of the cGMP/NO signaling pathway that operates during ABA-induced stomatal closure since our results show that it binds CNs and it has previously been shown to be S-nitrosylated [83] and regulate stomatal closure through HCO_3_^−^ effects on SLAC1 [106, 107].

Similarly, we propose that the expression correlated CNBP candidates could be direct targets of the pathogen-induced cGMP/NO signaling pathway. In support of this, the expression correlated CNBP candidates are annotated to function in the defence response, specifically incompatible interactions and the HR (Table 4). Additionally, their expression is repressed in response to virulent bacterial pathogens, *Psm* and *Pst* DC3000, as well as in response to the non-pathogenic *Pst* DC3000 *hrpA* mutant, supporting the annotated function in plant defence and suggesting that plants down regulate the expression of these genes as part of pathogen associated molecular pattern (PAMP) triggered immunity (Fig. 4). Finally, mutant studies provide further evidence that CA1, GOX1 and SHMT1 function in the defence response. Specifically *ca1* mutants are compromised in their defence against avirulent *Pst* DC3000 *avrB*; *gox1* mutants are compromised in their defence against virulent *Pst* DC3000 and non-host *P. syringae* pv *syringae* and *P. syringae* pv *tabaci* and *shmt1* mutants and compromised in their defence against virulent *Pst* DC3000 and avirulent *Pst* DC3000 *AvrRpm1* [70, 83, 108]. The compromised defence response phenotypes of these mutants may be due to defects in their H_2_O_2_ levels as *gox1* mutants produce less H_2_O_2_ and *shmt1* mutants produce more H_2_O_2_ in response to stress.

Here we showed that GOX activity is induced by avirulent *Pst* DC3000 *AvrRpm1* and this response is inhibited by a combination of NO and cGMP treatment (Fig. 5). It was somewhat surprising that neither NO nor cGMP alone was able to inhibit GOX activity because NO has been shown to induce cGMP synthesis in plants [10, 12, 15]. This could suggest that there are NO-mediated cGMP-independent pathways required for inhibition of GOX activity, for example NO-induced PTM [104]. Similarly, there may be cGMP pathways that are independent of upstream NO signalling that are required for inhibition of GOX activity. For example, cGMP synthesis via ligand-stimulated particulate GCs may be required for a full cGMP response to avirulent *Pst* DC3000 *AvrRpm1* and in support of this a leucine rich repeat containing Toll-like receptor has recently been shown to contain an active GC domain [109]. Therefore, NO-mediated cGMP-dependent and cGMP-independent pathways as well as cGMP pathways that are independent of NO may be required for inhibition of GOX activity.

The finding that *Pst* DC3000 *AvrRpm1*-induced GOX activity was inhibited by NO and cGMP supports that NO and cGMP signalling pathways can converge to modify GOX1 activity, and thus H_2_O_2_ production under conditions that elicit a HR. There are conflicting reports that place NO upstream and downstream of H_2_O_2_ in the plant response to pathogens [110] and there is evidence that cGMP stimulates H_2_O_2_ production [111, 112] although whether or not this happens in response to pathogens remains to be determined. We speculate that NO and cGMP inhibition of GOX activity is a negative feedback mechanism to turn off the H_2_O_2_ signal and prevent uncontrolled cell death during the HR. In support of this, NO-mediated PTM has been shown to inhibit NADPH oxidase activity [113] and stimulate ascorbate peroxidase activity [114], the combined effect of which would reduce H_2_O_2_ levels and this has been suggested to act as a negative feedback loop to limit the HR. This does not exclude the possibility that NO and cGMP stimulate H_2_O_2_ production at earlier time points in the response of Arabidopsis to avirulent *Pst* DC3000 *AvrRpm1*.

While we do not know how NO and cGMP affect the activity of the other CNBP candidates, we speculate that NO and cGMP can also modify their activities to fine-tune H_2_O_2_ signaling during the defence response.

## Conclusions

In conclusion, the Calvin cycle (PKG, GAPB and TKL) and photorespiratory enzymes (GOX1 and SHMT1) and the associated CA1 contain CNBD-like sequences and are modified by NO. Expression of these genes is correlated and they have GO annotations that suggest they function in H_2_O_2_ signaling the defence response. We have demonstrated that cGMP and NO treatment can modify the activity of at least one of the CNBP candidates, GOX1, that produces H_2_O_2_ in response to *Pst* DC3000 *AvrRpm1*. Therefore, the identified CNBP candidates have plausible roles in plant CN-mediated processes and we propose that they function together as points of cross-talk between CN, NO and H_2_O_2_ signaling during the defence response.

## Ethics approval and consent to participate

Not applicable

## Consent for publication

Not applicable

## Availability of data and materials

The datasets supporting the conclusions of this article are included within the article and its additional files.

## Additional files

Additional file 1: Experimental design to affinity purify CNBPs from Arabidopsis. (PDF 101 kb) Additional file 2: Arabidopsis proteins affinity purified by CN baits following negative subtraction. (PDF 113 kb) Additional file 3: Peptides identified in Arabidopsis CNBP candidates. (PDF 114 kb) Additional file 4: Alignment of CNBP candidates with known CNB domains. (PDF 242 kb) Additional file 5: Alignment of CNBP candidates with known GAF domains. (PDF 101 kb)

## Abbreviations

ATPD: atp synthase delta-subunit
CA1: carbonic anhydrase 1
cAMP: adenosine 3′, 5′-cyclic monophosphate
cGMP: guanosine 3′, 5′-cyclic monophosphate
CLPC1: caseinolytic protease c homolog 1
CN: cyclic nucleotide
CNB: cyclic nucleotide binding
CNBD: cyclic nucleotide binding domain
CNBP: cyclic nucleotide binding protein
CNGC: cyclic nucleotide-gated channel
CSP41B: chloroplast stem-loop binding protein of 41 kDa
EIF4A1: eukaryotic translation initiation factor 4A1
EPAC: exchange protein activated by cAMP
GAF: cGMP binding PDEs, *Anabaena* adenylyl cyclase and *E. coli* FhlA
GAPB: glyceraldehyde-3-phosphate dehydrogenase b subunit
GC: guanylyl cyclase
GO: gene ontology
GOX1: glycolate oxidase
H_2_O_2_: hydrogen peroxide
HCN: hyperpolarization-activated CN-modulated channel
HR: hypersensitive response
MS: mass spectrometry
NC: nucleotidyl cyclase
NO: nitric oxide
PDE: phosphodiesterase
PGK1: phosphoglycerate kinase 1
PKA: cAMP-dependent protein kinase
PKG: cGMP-dependent protein kinase
PORB: protochlorophyllide oxidoreductase B
*Psm*: *Pseudomonas**syringae* pv *maculicola*
*Pst*: *Pseudomonas**syringae* pv *tomato*
PTM: post-translationally modified
RABE1b: ras-related in brain GTPase homolog E1b
ROS: reactive oxygen species
SHMT1: serine hydroxymethyltransferase 1
TKL: transketolase
