# Supplementary material for: The arabidopsis cyclic nucleotide interactome
Source: Cell Commun Signal. 2016 May 11;14:10. doi: 10.1186/s12964-016-0133-2 (PMC4865018; doi:10.1186/s12964-016-0133-2)
Supplement: Additional file 1: — Experimental design to affinity purify CNBPs from Arabidopsis. (PDF 101 kb) [file 12964_2016_133_MOESM1_ESM.pdf]

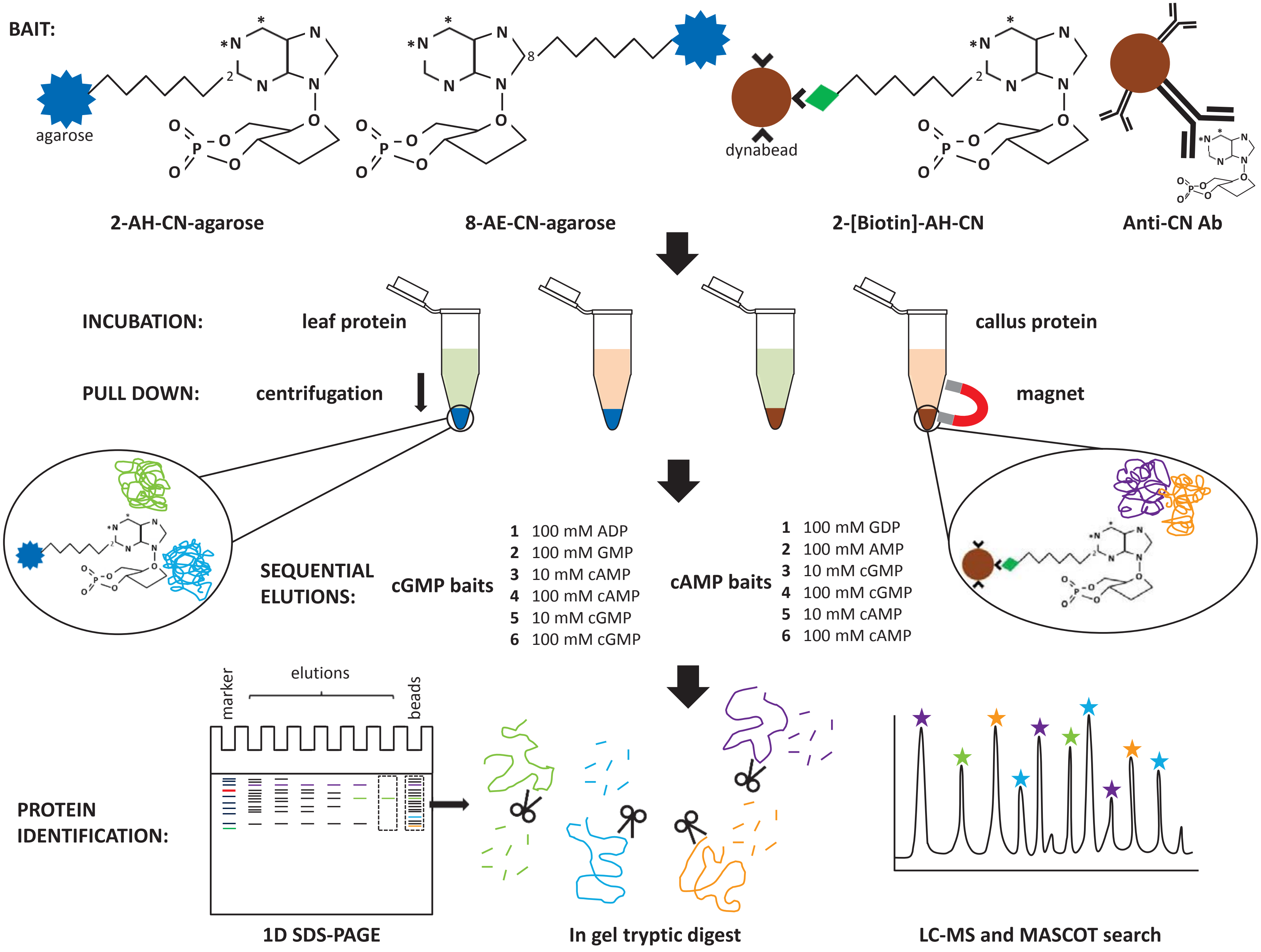
BAIT:
agarose
2-AH-CN-agarose
8-AE-CN-agarose
dynabead
2-[Biotin]-AH-CN
Anti-CN Ab
INCUBATION:
leaf protein
callus protein
PULL DOWN:
centrifugation
magnet
SEQUENTIAL ELUTIONS:
cGMP baits
1 100 mM ADP
2 100 mM GMP
3 10 mM cAMP
4 100 mM cAMP
5 10 mM cGMP
6 100 mM cGMP
cAMP baits
1 100 mM GDP
2 100 mM AMP
3 10 mM cGMP
4 100 mM cGMP
5 10 mM cAMP
6 100 mM cAMP
PROTEIN IDENTIFICATION:
marker
elutions
beads
1D SDS-PAGE
In gel tryptic digest
LC-MS and MASCOT search

Additional file 1. Experimental design to affinity purify cyclic nucleotide binding proteins from Arabidopsis

The four different types of baits are depicted with * indicating positions at which cAMP differs from cGMP. The agarose baits are indicated by blue flashes while the magnetic dynabead baits are represented as brown circles. Leaf (pale green) or callus (pale pink) extracts were incubated with the baits then pulled down using centrifugation or a Dynamag magnet depending on the type of bait. Interactions that formed were then challenged with an increasing stringent sequential elution process. Elution and bead fractions were separated by one dimensional (ID) SDS-PAGE, the lanes excised and proteins digested with trypsin. Peptide fragments were detected by nano-liquid chromatography (LC)/MS and matched to the Arabidopsis_TAIR10 protein database using MASCOT.
