# Supplementary material for: The arabidopsis cyclic nucleotide interactome
Source: Cell Commun Signal. 2016 May 11;14:10. doi: 10.1186/s12964-016-0133-2 (PMC4865018; doi:10.1186/s12964-016-0133-2)
Supplement: Additional file 2: — Arabidopsis proteins affinity purified by CN baits following negative subtraction. (PDF 113 kb) [file 12964_2016_133_MOESM2_ESM.pdf]

Additional file 2. Arabidopsis proteins affinity purified with cyclic nucleotide baits following negative subtraction

		LEAF cAMP				LEAF cGMP				CALLUS cAMP				CALLUS cGMP			
**DESCRIPTION**	**ACCESSION**	2-AHA-cAMP-agarose	8-AEA-cAMP-agarose	2-[Biotin]-AHA-cAMP	cAMP antibody	2-AH-cGMP-agarose	8-AET-cGMP-agarose	2-[Biotin]-AH-cGMP	cGMP antibody	2-AHA-cAMP-agarose	8-AEA-cAMP-agarose	2-[Biotin]-AHA-cAMP	cAMP antibody	2-AH-cGMP-agarose	8-AET-cGMP-agarose	2-[Biotin]-AH-cGMP	cGMP antibody
Nucleoside diphosphate kinase family protein (NDPK1)	AT4G09320		1,2,3,4,B		B	1,2,3,4,5,6,B	1,2,3,4,5,6,B			1,2,3,B	1,2,3,B			1,2,3,4,5,6,B	1,2,3,4,5,6,B		
Nucleoside diphosphate kinase 3 (NDPK3)	AT4G11010		1,2,3,4,5			1,2,3,4	1,2,3,B			1				2,3	1,2,3		
Serine transhydroxymethyltransferase 1 (SHMT1)	AT4G37930	2,B	B	1,B	B	B	B	2,3,B									
Glyceraldehyde-3-phosphate dehydrogenase B subunit (GAPB)	AT1G42970	B	B	B	1,2,B	B	B	B	B								
NAD(P)-binding Rossmann-fold superfamily protein	AT2G47130													2,3,4,5			
Phosphoglycerate kinase 1 (PGK1)	AT3G12780	B	B	B	B	B	B	B	B								
Ribosomal protein L6 family	AT1G33120 AT1G33140									1,2,3,4,5,B	1,3,4						
Eukaryotic translation initiation factor 4A1 (EIF4A1)	AT3G13920			B		B			B	B	B	B	B	B	B	B	B
Breast basic conserved 1 (ATBBC1)	AT3G49010	4,B	1							1,2,3,4,B	1		2				1
Ribosomal protein S8e family protein	AT5G20290					B				1,2,3,4,6,B	1			B			
RAB GTPase homolog E1B (ATRABE1B)	AT4G20360	B	B	B	1,2,B	B	B	B	B								
Nucleoside diphosphate kinase 2 (NDPK2)	AT5G63310						1,2,3,5,B								1		
Ribosomal protein L5 (RPL5A)	AT3G25520									1,2,4,5,B	1						
Ribosomal protein S5 domain 2-like superfamily protein	AT2G09990									1,3	1						
Transketolase	AT3G60750		B	B	B	B	B	B	B								
Cytosolic ribosomal protein S15 (RPS15)	AT1G04270									1,2,4,B							
Chloroplast RNA binding (CSP41B)	AT1G09340			1	B	B		2,B									

Ribosomal protein S26e family protein	AT2G40510<br>AT2G40590<br>AT3G56340									1,2,3,4,6,B	1						
Auxin transport protein (BIG)	AT3G02260															B	
Ribosomal protein S13A (ATRPS13A)	AT4G00100	3,B								1,2,3,4	1						
Ribosomal protein S5 family protein	AT2G41840									1,2	1						
CLPC homologue 1 (CLPC1)	AT5G50920	B	B	B	B	B	B	B									
Metallopeptidase M24 family protein (ATEBP1)	AT3G51800									1,2,3,4,6	1						
Ribosomal protein L14p/L23e family protein	AT1G04480<br>AT2G33370<br>AT3G04400									1,2,B	1,2						
Ribosomal protein L18ae/LX family protein	AT2G34480									1	4			B			
Ribosomal protein L23/L15e family protein	AT4G16720<br>AT4G17390									1,2,4,5,6	1,4						
Translation protein SH3-like family protein	AT3G49910	3								1,4,B	1						
Ribosomal protein L32e	AT4G18100									1,2,B	1						
Glucoside glucohydrolase 2 (BGLU37)	AT5G25980		1	1													
Ribosomal protein L24e family protein (RPL24B)	AT3G53020									1	1						
Zinc-binding ribosomal protein family protein	AT3G60245									1	1						
Ribosomal protein L6 family protein	AT1G18540									1,2,3,6	1						
Ribosomal protein L22p/L17e family protein	AT1G67430									1,3,B	1						
Ribosomal protein L36e family protein	AT3G53740									1			2				
Subunit of exocyst complex 8 (ATSEC8)	AT3G10380															B	
Aldolase-type TIM barrel family protein	AT3G14420		B		B			B	B								
Ribosomal protein L7Ae/L30e/S12e/Gadd45 family protein	AT3G18740									6,B							
Tubulin beta-7 chain (TUB7)	AT2G29550									B							
Ribosomal protein L12-A (RPL12-A)	AT3G27830<br>AT3G27850	3,B	1		3												
Ribosomal protein L13 family protein	AT3G24830									1							
acetyl-CoA carboxylase 1 (ACC1)	AT1G36160											B				B	
Ribosomal L29 family protein	AT3G09500									1,B							
Ribosomal protein L7Ae/L30e/S12e/Gadd45 family protein	AT1G15930									1	1						

Ribosomal protein S6 (RPS6A)	AT4G31700									1							
60S acidic ribosomal protein family	AT2G27710									1,2,3,6	1,2						
Catalase 2 (CAT2)	AT4G35090					2,B											
Glutamine synthase clone F11 (ATGSR2)	AT1G66200									1							
Chloroplast stem-loop binding protein of 41 kDa (CSP41A)	AT3G63140			1,B				B									
Ribosomal protein L19e family protein (emb2386)	AT1G02780									1,2	1						
Ribosomal protein L30/L7 family protein	AT2G44120									1,B							
Secretion-associated RAS 1B (SAR1B)	AT1G56330<br>AT3G62560<br>AT4G02080									B					B		B
Ribosomal protein L16p/L10e family protein (RPL10A)	AT1G14320										1						
Protochlorophyllide oxidoreductase B (PORB)	AT4G27440		B		B	B											
Ribosomal protein S24e family protein	AT3G04920									1							
Ribosomal protein L35Ae family protein	AT1G41880<br>AT3G55750									6							
Zinc-binding ribosomal protein family protein	AT2G45710<br>AT3G61110									B	1						
Alanine:glyoxylate aminotransferase (SGAT)	AT2G13360					B											
Ribosomal protein S25 family protein	AT4G39200									1							
Ribosomal L28e protein family	AT2G19730									1							
Ribosomal protein L3 family protein	AT2G43030	4,B															
Ribosomal protein S5/Elongation factor G/III/V family protein (LOS1)	AT1G56070									B							
Chloroplastic acetylcoenzyme A carboxylase 1 (CAC1A)	AT5G16390			B								B				B	
60S acidic ribosomal protein family	AT5G57290									1							
Serine hydroxymethyltransferase 4 (SHM4)	AT4G13930				B				B								
ATP synthase delta-subunit gene (ATPD)	AT4G09650			B		B		B	B								
Ribosomal protein S28 (RPS28)	AT5G64140										1						
Ribosomal protein L15 (RPL15)	AT3G25920	3,4<br>,B															
Ribosomal protein L4/L1 family	AT5G02870									1							

Polynucleotidyl transferase, ribonuclease H-like superfamily protein	AT3G11770									6					2		
TCP-1/cpn60 chaperonin family protein	AT3G11830											1				2	
Coatomer gamma-2 subunit, putative / gamma-2 coat protein, putative / gamma-2 COP, putative	AT4G34450									1							
Ribosomal protein S7p/S5e family protein (RPS7.1)	ATCG00900 ATCG01240	3,B			B												
Ribosomal protein S7e family protein	AT1G48830									1							
ATPase, F1 complex, gamma subunit protein (ATPC1)	AT4G04640	B															
Adenylosuccinate synthase (ADSS)	AT3G57610					B											
Glutamine synthetase 1.3 (ATGSKB6)	AT3G17820									1							
ADP/ATP carrier 3 (ATAAC3)	AT4G28390											B					
Carbonic anhydrase 1 (CA1)	AT3G01500			B		B											
Chlorophyll A/B binding protein 3 (CAB3)	AT1G29910 AT1G29920 AT1G29930 AT2G34420				B		5										
Methylesterase PCR A (ATPMEPCRA)	AT1G11580									6							
Xyloglucan endotransglucosylase/hydrolase 4 (XTH4)	AT2G06850		1														
Unknown protein	AT2G15860												B				B
Single hybrid motif superfamily protein	AT1G52670			B													
Isocitrate dehydrogenase 1 (IDH1)	AT4G35260												B				
Histone superfamily protein (H2B)	AT3G45980	3															
Ras-related small GTP-binding family protein	AT5G59840												B				B
UBX domain-containing protein	AT4G10790																B
Regulatory particle triple-A ATPase 5A (RPT5A)	AT3G05530																B
Polynucleotidyl transferase, ribonuclease H-like superfamily protein	AT5G06450									6	1						
Ribosomal protein L9 (RPL9)	AT3G44890	B															
Ribosomal protein L6	AT1G05190	4															

family (emb2394)																	
Actin 1 (ACT1)	AT2G37620 AT3G53750																B
Tubulin beta-5 chain (TUB5)	AT1G20010				B												
Ribosomal L5P family protein	AT4G01310	B															
Guanylate-binding family protein	AT5G46070					2,B											
3-deoxy-D-arabino-heptulosonate 7-phosphate synthase 1 (DHS1)	AT4G39980												B				
Plastid-lipid associated protein PAP / fibrillin family protein	AT2G35490																B
TCP-1/cpn60 chaperonin family protein	AT3G03960											1					
Photosystem II subunit Q-2 (PSBQ-2)	AT4G05180							B									
Biotin/lipoyl attachment domain-containing protein	AT3G56130											B					
Ribosomal protein S9 (RPS9)	AT1G74970	3															
GAST1 protein homolog 1 (GASA1)	AT1G75750															B	
ARM repeat superfamily protein	AT3G62530				B												
Ribosomal protein L17 family protein	AT3G54210	B															
Tubulin beta-9 chain (TUB9)	AT4G20890																B
Adenine nucleotide alpha hydrolases-like superfamily protein	AT3G53990																B
Ribosomal protein S19e family protein	AT5G61170									1							
Eukaryotic aspartyl protease family protein	AT3G54400							1									
Coatomer epsilon subunit	AT1G30630									B							
Fibrillin (FIB)	AT4G04020								B				B				
RAB GTPase homolog G3E (ATRABG3E)	AT1G49300												B				
Aluminium induced protein with YGL and LRDR motifs	AT4G27450									B							
NAD(P)-binding Rossmann-fold superfamily protein	AT1G24360	4															
Chloroplast 30S ribosomal protein S20, putative	AT3G15190	3															
RAB GTPase homolog 1C (ATRAB1C)	AT4G17530 AT5G47200																B

SHK1 binding protein 1 (SKB1)	AT4G31120													2			
Glycosyl hydrolase superfamily protein (BGLU22)	AT1G66280													2			
Ribosomal protein S13/S18 family	AT5G14320	B															
Ribosomal protein S18 (RPS18)	ATCG00650	B															

The fraction in which the protein was identified is indicated with numbers 1-6 being the elution fractions (1 denotes the first and least stringent elution and 6 the final and most stringent elution) while B indicates that the protein was retained on the bead. For the cAMP baits the elutions were 1) 100 mM GDP; 2) 100 mM AMP; 3) 10 mM cGMP; 4) 100 mM cGMP; 5) 10 mM cAMP and 6) 100 mM cAMP. For the cGMP baits the elutions were 1) 100 mM ADP; 2) 100 mM GMP; 3) 10 mM cAMP; 4) 100 mM cAMP; 5) 10 mM cGMP and 6) 100 mM cGMP.
