# Supplementary material for: The arabidopsis cyclic nucleotide interactome
Source: Cell Commun Signal. 2016 May 11;14:10. doi: 10.1186/s12964-016-0133-2 (PMC4865018; doi:10.1186/s12964-016-0133-2)
Supplement: Additional file 3: — Peptides identified in Arabidopsis CNBP candidates. (PDF 114 kb) [file 12964_2016_133_MOESM3_ESM.pdf]

Additional file 3. Peptides identified in the Arabidopsis cyclic nucleotide binding protein candidates

Protein	MW (Da)	Experiment	Bait	Fract	Protein ID prob	# uniq peptide	# total spectra	% seq cover	Peptide sequence	Peptide ID prob
AT3G13920	46,704.90	LEAF cAMP	2-[Biotin]-AHA-cAMP	bead	99.8%	2	2	7.77%	GLDVIQQAQSGTGK	95.0%
									SRDHTVSATHGDMDQNTR	95.0%
		LEAF cGMP	2-AH-cGMP-agarose	bead	100.0%	2	3	5.34%	ELAQQIEK	95.0%
									GLDVIQQAQSGTGK	95.0%
			cGMP antibody	bead	100.0%	3	4	10.40%	DHTVSATHGDMDQNTR	95.0%
									GLDVIQQAQSGTGK	95.0%
									MFVLDEADEMLSR	95.0%
		CALLUS cAMP	2-AHA-cAMP-agarose	bead	100.0%	6	9	16.70%	ALGDYLGVK	95.0%
									ELAQQIEK	95.0%
									FYNVVVEELPSNVADLL	95.0%
									GLDVIQQAQSGTGK	95.0%
									KVDWLTDK	95.0%
									MFVLDEADEMLSR	95.0%
			8-AEA-cAMP-agarose	bead	100.0%	3	5	7.77%	ELAQQIEK	95.0%
									GLDVIQQAQSGTGK	95.0%
									VLITTDLLAR	95.0%
			2-[Biotin]-AHA-cAMP	bead	100.0%	3	5	8.98%	GLDVIQQAQSGTGK	95.0%
									MFVLDEADEMLSR	95.0%
									VLITTDLLAR	95.0%
			cAMP antibody	bead	100.0%	2	4	6.55%	GLDVIQQAQSGTGK	95.0%
									MFVLDEADEMLSR	95.0%
		CALLUS cGMP	2-AH-cGMP-agarose	bead	100.0%	2	5	7.52%	MFVLDEADEMLSR	95.0%
									SRDHTVSATHGDMDQNTR	95.0%
			8-AET-cGMP-agarose	bead	100.0%	5	6	12.90%	ELAQQIEK	95.0%
									GLDVIQQAQSGTGK	95.0%
									KVDWLTDK	95.0%
									MFVLDEADEMLSR	95.0%
									VLITTDLLAR	95.0%
			2-[Biotin]-AH-cGMP	bead	100.0%	5	9	15.30%	ELAQQIEK	94.7%
									GLDVIQQAQSGTGK	95.0%
									MFVLDEADEMLSR	95.0%
									SRDHTVSATHGDMDQNTR	95.0%
									VLITTDLLAR	95.0%
			cGMP antibody	bead	100.0%	4	8	12.40%	DHTVSATHGDMDQNTR	95.0%
									ELAQQIEK	95.0%
									GLDVIQQAQSGTGK	95.0%
									MFVLDEADEMLSR	95.0%
AT3G12780	50,113.10	LEAF cAMP	2-AHA-cAMP-agarose	bead	100.0%	3	3	8.32%	ELDYLVGAVSNPK	95.0%
									FSLAPLVPR	95.0%
									LASLADLYVNDAFGTAHR	95.0%
				bead	100.0%	4	4	10.80%	ELDYLVGAVSNPK	95.0%
									FLKPSVAGFLLQK	95.0%
									FSLAPLVPR	95.0%
									GVTTIIGGGDSVAAVEK	95.0%
			2-[Biotin]-AHA-cAMP	bead	100.0%	5	8	14.30%	ELDYLVGAVSNPK	95.0%
									FAAGTEAIANK	95.0%
									FSLAPLVPR	95.0%
									GVTTIIGGGDSVAAVEK	95.0%
									KLASLADLYVNDAFGTAHR	95.0%
			cAMP antibody	bead	100.0%	9	12	22.50%	AAIPTIK	95.0%

									AQGLSVGSSLVEEDKLELATELLAK	95.0%
									ELDYLVGAVSNPK	95.0%
									FAAGTEAIANK	95.0%
									FSLAPLVPR	95.0%
									GVTTIIGGGDSVAAVEK	95.0%
									KLASLADLYVNDAFGTAHR	95.0%
									LAELSEK	95.0%
									LASLADLYVNDAFGTAHR	95.0%
		LEAF cGMP	2-AH-cGMP-agarose	bead	100.0%	3	5	9.36%	AAIPTIK	95.0%
									AQGLSVGSSLVEEDKLELATELLAK	95.0%
									ELDYLVGAVSNPK	95.0%
			8-AET-cGMP-agarose	bead	100.0%	2	5	4.57%	ELDYLVGAVSNPK	95.0%
									FSLAPLVPR	95.0%
			2-[Biotin]-AH-cGMP	bead	100.0%	8	12	18.10%	AAIPTIK	95.0%
									ELDYLVGAVSNPK	95.0%
									FAAGTEAIANK	95.0%
									FSLAPLVPR	95.0%
									GVTTIIGGGDSVAAVEK	95.0%
									KLASLADLYVNDAFGTAHR	95.0%
									LASLADLYVNDAFGTAHR	95.0%
									SVGDLTSADLK	95.0%
			cGMP antibody	bead	100.0%	8	9	18.50%	AAIPTIK	95.0%
									ELDYLVGAVSNPK	95.0%
									FLKPSVAGFLLQK	95.0%
									FSLAPLVPR	95.0%
									GVTTIIGGGDSVAAVEK	95.0%
									KLASLADLYVNDAFGTAHR	95.0%
									LASLADLYVNDAFGTAHR	95.0%
									SVGDLTSADLK	95.0%
AT1G42970	47,659.30	LEAF cAMP	2-AHA-cAMP-agarose	bead	100.0%	4	4	20.40%	AVSLVLPQLK	95.0%
									DSPLEVVVLNDSGGVK	95.0%
									IVDNETISVDGK	95.0%
									VIITAPAK	95.0%
			8-AEA-cAMP-agarose	bead	100.0%	6	6	20.10%	AVSLVLPQLK	95.0%
									GLTAEDVNEAFR	95.0%
									GLTAEDVNEAFRK	94.9%
									IVDNETISVDGK	95.0%
									VIITAPAK	95.0%
									VLDEEFGIVK	95.0%
			2-[Biotin]-AHA-cAMP	bead	100.0%	6	9	20.60%	AVSLVLPQLK	95.0%
									GLTAEDVNEAFR	95.0%
									IVDNETISVDGK	95.0%
									VIITAPAK	95.0%
									VLDEEFGIVK	95.0%
									VVDLAHLVASK	95.0%
			cAMP antibody	AMP	99.9%	2	3	12.80%	IVDNETISVDGK	95.0%
									VIITAPAK	95.0%
				10 cGMP	99.8%	2	2	6.94%	VIITAPAK	95.0%
									YDSMLGTFK	95.0%
				bead	100.0%	10	18	32.20%	AVSLVLPQLK	95.0%
									DSPLEVVVLNDSGGVK	95.0%
									GLTAEDVNEAFR	95.0%
									GLTAEDVNEAFRK	95.0%
									IVDNETISVDGK	95.0%
									VIITAPAK	95.0%

									VLDEEFGIVK	95.0%
									VPTPNVSVVDLVINVEKK	95.0%
									VVDLAHLVASK	95.0%
									YDSMLGTFK	95.0%
		LEAF cGMP	2-AH-cGMP-agarose	bead	100.0%	6	11	23.30%	AVSLVLPQLK	95.0%
									DSPLEVVVLNDSGGVK	95.0%
									GLTAEDVNEAFR	95.0%
									IVDNETISVDGK	95.0%
									VIITAPAK	95.0%
									VLDEEFGIVK	95.0%
			8-AET-cGMP-agarose	bead	100.0%	5	9	21.50%	AVSLVLPQLK	95.0%
									DSPLEVVVLNDSGGVK	95.0%
									IVDNETISVDGK	95.0%
									VLDEEFGIVK	95.0%
									VVDLAHLVASK	95.0%
			2-[Biotin]-AH-cGMP	bead	100.0%	8	10	28.20%	AVSLVLPQLK	95.0%
									DSPLEVVVLNDSGGVK	95.0%
									GLTAEDVNEAFR	95.0%
									IVDNETISVDGK	95.0%
									VIITAPAK	95.0%
									VLDEEFGIVK	95.0%
									VPTPNVSVVDLVINVEKK	95.0%
									VVDLAHLVASK	95.0%
			cGMP antibody	bead	100.0%	7	12	23.70%	AVSLVLPQLK	95.0%
									GLTAEDVNEAFR	95.0%
									IVDNETISVDGK	95.0%
									VIITAPAK	95.0%
									VLDEEFGIVK	95.0%
									VVAWYDNEWGYSQR	95.0%
									VVDLAHLVASK	95.0%
AT4G20360	51,630.70	LEAF cAMP	2-AHA-cAMP-agarose	bead	100.0%	4	4	12.40%	ILDEALAGDNVGLLLR	95.0%
									SYTVTGVEMFQK	95.0%
									TTLTAALTMALASIGSSVAK	95.0%
									VGETVDLVGLR	95.0%
			8-AEA-cAMP-agarose	bead	100.0%	3	4	7.35%	KYDEIDAAPEER	95.0%
									SYTVTGVEMFQK	95.0%
									VGETVDLVGLR	95.0%
			2-[Biotin]-AHA-cAMP	bead	100.0%	4	7	12.20%	ILDEALAGDNVGLLLR	95.0%
									KYDEIDAAPEER	95.0%
									QTELPFLLAVEDVFSITGR	95.0%
									VGETVDLVGLR	95.0%
			cAMP antibody	AMP	100.0%	3	3	8.19%	GITINTATVEYETENR	95.0%
									SYTVTGVEMFQK	95.0%
									VGETVDLVGLR	95.0%
				10 cGMP	100.0%	3	3	8.19%	GITINTATVEYETENR	95.0%
									SYTVTGVEMFQK	95.0%
									VGETVDLVGLR	95.0%
				bead	100.0%	7	9	21.40%	EHILLAK	95.0%
									GITINTATVEYETENR	95.0%
									ILDEALAGDNVGLLLR	95.0%
									KYDEIDAAPEER	95.0%
									QTELPFLLAVEDVFSITGR	95.0%
									SYTVTGVEMFQK	95.0%
									TTLTAALTMALASIGSSVAK	95.0%
		LEAF cGMP	2-AH-cGMP-agarose	bead	100.0%	4	9	11.60%	GITINTATVEYETENR	95.0%

									ILDEALAGDNVGLLLR	95.0%
									KYDEIDAAPEER	95.0%
									VGETVDLVGLR	95.0%
			8-AET-cGMP-agarose	bead	100.0%	4	5	12.40%	ILDEALAGDNVGLLLR	95.0%
									KYDEIDAAPEER	95.0%
									TTLTAALTMALASIGSSVAK	95.0%
									VGETVDLVGLR	95.0%
			2-[Biotin]-AH-cGMP	bead	100.0%	3	6	8.19%	ILDEALAGDNVGLLLR	95.0%
									KYDEIDAAPEER	95.0%
									VGETVDLVGLR	95.0%
			cGMP antibody	bead	100.0%	4	5	10.70%	ILDEALAGDNVGLLLR	95.0%
									KYDEIDAAPEER	95.0%
									SYTVTGVEMFQK	95.0%
									VGETVDLVGLR	95.0%
AT3G60750	79,970.10	LEAF cAMP	8-AEA-cAMP-agarose	bead	100.0%	7	7	9.99%	FLAIDAVEK	95.0%
									HTPEGATLESDWSAK	95.0%
									KYPEEASELK	95.0%
									NLSQQCLNALAK	95.0%
									NPYWFNR	95.0%
									TPSILALSR	95.0%
									VTTTIGYGSPNK	95.0%
			2-[Biotin]-AHA-cAMP	bead	100.0%	3	3	5.40%	ALPTYTPESPGDATR	94.5%
									SIITGELPAGWEK	95.0%
									VTTTIGYGSPNK	95.0%
			cAMP antibody	bead	100.0%	3	3	5.80%	AMPNTLMFRPADGNETAGAYK	95.0%
									KYPEEASELK	95.0%
									NLSQQCLNALAK	95.0%
		LEAF cGMP	2-AH-cGMP-agarose	bead	100.0%	7	7	11.30%	AMPNTLMFRPADGNETAGAYK	95.0%
									ANSYSVHGAALGEK	95.0%
									ANSYSVHGAALGEKEVEATR	95.0%
									FLAIDAVEK	95.0%
									NLSQQCLNALAK	95.0%
									SIITGELPAGWEK	95.0%
									TPSILALSR	95.0%
			8-AET-cGMP-agarose	bead	100.0%	3	3	5.53%	KYPEEASELK	95.0%
									SIGINSFGASAPAPLLYK	95.0%
									SIITGELPAGWEK	95.0%
			2-[Biotin]-AH-cGMP	bead	100.0%	8	9	12.00%	ANSYSVHGAALGEK	95.0%
									ANSYSVHGAALGEKEVEATR	95.0%
									FLAIDAVEK	95.0%
									NLSQQCLNALAK	95.0%
									SIGINSFGASAPAPLLYK	95.0%
									TPSILALSR	95.0%
									VTTTIGYGSPNK	95.0%
									YPEEASELK	94.9%
			cGMP antibody	bead	100.0%	3	3	4.99%	NLSQQCLNALAK	95.0%
									SIITGELPAGWEK	95.0%
									VTTTIGYGSPNK	95.0%
AT5G50920	103455.6	LEAF cAMP	2-AHA-cAMP-agarose	bead	99.8%	2	2	2.48%	HIEKDPALER	95.0%
									VLENLGADPSNIR	95.0%
			8-AEA-cAMP-agarose	bead	100.0%	4	4	4.63%	AEVSAIQAK	95.0%
									AIDLIDEAGSR	95.0%
									TAIAEGLAQR	95.0%
									VLENLGADPSNIR	95.0%
			2-[Biotin]-AHA-cAMP	bead	100.0%	3	3	3.55%	AIDLIDEAGSR	95.0%

									HAQVPEEAR	95.0%
									VLENLGADPSNIR	95.0%
			cAMP antibody	bead	100.0%	3	3	3.66%	AIDLIDEAGSR	95.0%
									TAIAEGLAQR	95.0%
									VLENLGADPSNIR	95.0%
		LEAF cGMP	2-AH-cGMP-agarose	bead	100.0%	3	3	3.23%	LLEDSMAEK	94.8%
									SLVTEELK	95.0%
									VLENLGADPSNIR	95.0%
			8-AET-cGMP-agarose	bead	100.0%	4	4	5.06%	GSGFVAVEIPFTPR	95.0%
									HIEKDPALER	95.0%
									TAIAEGLAQR	95.0%
									VLENLGADPSNIR	94.6%
			2-[Biotin]-AH-cGMP	bead	100.0%	4	4	5.60%	LDMSEFMER	95.0%
									MVGENNEVTANVGGGSSSNK	95.0%
									TAIAEGLAQR	95.0%
									VLENLGADPSNIR	95.0%
AT4G37930	57,401.70	LEAF cAMP	2-AHA-cAMP-agarose	AMP	99.8%	2	2	3.68%	FAQTLMER	95.0%
									LIVAGASAYAR	95.0%
				bead	100.0%	3	3	6.96%	LIVAGASAYAR	95.0%
									NTVPGDVSAMVPGGIR	95.0%
									YSEGYPGAR	95.0%
			8-AEA-cAMP-agarose	bead	100.0%	5	6	9.48%	AYQEQVLSNSAK	95.0%
									FAQTLMER	95.0%
									MGTPALTSR	95.0%
									VLEAVHIASNK	95.0%
									YSEGYPGAR	95.0%
			2-[Biotin]-AHA-cAMP	GDP	100.0%	8	17	12.80%	AYQEQVLSNSAK	95.0%
									FAQTLMER	95.0%
									GAMIFFR	95.0%
									HEVEEFAK	95.0%
									LRHEVEEFAK	95.0%
									MGTPALTSR	95.0%
									VLEAVHIASNK	95.0%
									YSEGYPGAR	95.0%
				bead	100.0%	6	6	15.70%	GYELVSGGTDNHLVLVNLKPK	95.0%
									LRHEVEEFAK	95.0%
									MGTPALTSR	95.0%
									QLNAPLEEVDPEIADIIEHEK	95.0%
									VLEAVHIASNK	95.0%
									YSEGYPGAR	95.0%
			cAMP antibody	bead	100.0%	3	3	6.00%	AYQEQVLSNSAK	95.0%
									LRHEVEEFAK	95.0%
									YSEGYPGAR	95.0%
		LEAF cGMP	2-AH-cGMP-agarose	bead	100.0%	4	4	12.00%	LKDFVSAMESSSTIQSEIAK	95.0%
									LRHEVEEFAK	95.0%
									QLNAPLEEVDPEIADIIEHEK	95.0%
									VLEAVHIASNK	95.0%
			8-AET-cGMP-agarose	bead	100.0%	3	3	6.96%	AYQEQVLSNSAK	95.0%
									FAQTLMER	95.0%
									NTVPGDVSAMVPGGIR	94.9%
			2-[Biotin]-AH-cGMP	GMP	100.0%	6	9	12.20%	AYQEQVLSNSAK	95.0%
									FAQTLMER	95.0%
									MGTPALTSR	95.0%
									NTVPGDVSAMVPGGIR	95.0%
									QFPTIGFEK	95.0%

									YSEGYPGAR	95.0%
				10 cAMP	100.0%	2	2	3.48%	MGTPALTSR	95.0%
									YSEGYPGAR	95.0%
				beads	100.0%	7	9	16.60%	AYQEQVLSNSAK	95.0%
									FAQTLMER	95.0%
									LKDFVSAMESSSTIQSEIAK	95.0%
									LRHEVEEFAK	95.0%
									NTVPGDVSAMVPGGIR	95.0%
									VLEAVHIASNK	95.0%
									YSEGYPGAR	95.0%
AT3G14420	39,463.60	LEAF cAMP	8-AEA-cAMP-agarose	bead	100.0%	3	3	8.33%	FFQLYVYK	95.0%
									FTLPPNLTLK	95.0%
									MAHPDGEYATAR	95.0%
			cAMP antibody	bead	100.0%	7	7	26.90%	AASAAGTIMTLSSWATSSVEEVASTGPGIR	95.0%
									AIALTVDTPR	95.0%
									GVLTGEDAR	95.0%
									IPVFLDGGVR	95.0%
									MAHPDGEYATAR	95.0%
									NFEGLDLGK	95.0%
									QLDYVPATISALEEVVK	95.0%
		LEAF cGMP	2-[Biotin]-AH-cGMP	bead	100.0%	2	2	6.11%	AIALTVDTPR	95.0%
									MAHPDGEYATAR	95.0%
			cGMP antibody	bead	100.0%	3	3	10.00%	AIALTVDTPR	95.0%
									GVLTGEDAR	95.0%
									QLDYVPATISALEEVVK	95.0%
AT4G09650	25,669.10	LEAF cAMP	2-[Biotin]-AHA-cAMP	bead	99.9%	2	2	9.83%	QVIDDIVK	95.0%
									SVVKLEAPQLAQIAK	95.0%
		LEAF cGMP	2-AH-cGMP-agarose	bead	100.0%	2	2	8.97%	LEAPQLAQIAK	95.0%
									LTDTQLAEVR	95.0%
			8-AET-cGMP-agarose	bead	100.0%	4	4	22.60%	LTDTQLAEVR	95.0%
									QVIDDIVK	95.0%
									SSSLQSHTSNFLNVLVDANR	95.0%
									SVVKLEAPQLAQIAK	95.0%
			2-[Biotin]-AH-cGMP	bead	99.8%	2	2	7.69%	LTDTQLAEVR	95.0%
									QVIDDIVK	95.0%
AT1G09340	42,621.40	LEAF cAMP	2-[Biotin]-AHA-cAMP	GDP	100.0%	7	10	22.50%	DLATAFLNVLGNEK	95.0%
									EADFTTDDMILSK	95.0%
									EAEEVEPILEALPK	95.0%
									EIFNISGEK	95.0%
									LETESLLQSK	95.0%
									QLPGESDQDFADFSSK	95.0%
									YVTFDGLAK	95.0%
			cAMP antibody	bead	100.0%	2	3	7.94%	DLATAFLNVLGNEK	95.0%
									QLPGESDQDFADFSSK	95.0%
		LEAF cGMP	2-AH-cGMP-agarose	bead	99.8%	2	4	7.41%	DLATAFLNVLGNEK	95.0%
									EAEEVEPILEALPK	95.0%
			2-[Biotin]-AH-cGMP	GMP	100.0%	2	3	7.14%	EADFTTDDMILSK	95.0%
									EAEEVEPILEALPK	95.0%
				bead	100.0%	3	5	9.79%	DLATAFLNVLGNEK	95.0%
									EAEEVEPILEALPK	95.0%
									EIFNISGEK	95.0%
AT1G56330<br>AT3G62560<br>AT4G02080	21,987.60	CALLUS cAMP	2-AHA-cAMP-agarose	bead	100.0%	3	3	14.50%	IDIPYAASEDELR	95.0%
									VDAVVYLVDAYDK	95.0%

									VDAVVYLVDAYDKER	95.0%
		CALLUS cGMP	8-AET-cGMP-agarose	bead	100.0%	2	3	9.84%	AFDLGGHQIAR	95.0%
									MGYGEGFK	95.0%
			cGMP antibody	bead	100.0%	3	3	20.20%	IDIPYAASEDELR	95.0%
									ILFLGLDNAGK	95.0%
									VDAVVYLVDAYDKER	95.0%
AT4G27440	43,360.50	LEAF cAMP	8-AEA-cAMP-agarose	bead	99.8%	2	2	7.73%	KGNVVVTGASSGLGLATAK	95.0%
									LAQVVSDPSLTK	95.0%
			cAMP antibody	bead	99.9%	2	2	7.98%	GYVSETESGKR	95.0%
									RTETPLDVLVCNAAVYFPTAK	95.0%
		LEAF cGMP	2-AH-cGMP-agarose	bead	99.8%	2	2	4.74%	ANLGDLR	95.0%
									LAQVVSDPSLTK	95.0%
AT3G01500	37,450.70	LEAF cAMP	2-[Biotin]-AHA-cAMP	bead	100.0%	3	3	20.70%	AFDPVETIK	95.0%
									VCPSHVLDFQPGDAFVVR	95.0%
									VISELGDSAFEDQCGR	95.0%
		LEAF cGMP	2-AH-cGMP-agarose	bead	99.8%	2	2	14.80%	AFDPVETIK	95.0%
									VISELGDSAFEDQCGR	95.0%

Peptide sequences identified in each of the Arabidopsis CNBP candidates. The molecular weight (MW) is given in Dalton (Da). The fraction (Fract) in which the protein was identified is indicated with the number showing the millimolar concentration of the nucleotide in the elution series. Probability (Prob) of protein identification (ID) and peptide ID was calculated using Scaffold. The number (#) of unique (uniq) peptides and the # of total spectra is shown. Percentage (%) sequence (seq) coverage (cover) reveals the proportion of the protein identified.
