# Supplementary material for: The arabidopsis cyclic nucleotide interactome
Source: Cell Commun Signal. 2016 May 11;14:10. doi: 10.1186/s12964-016-0133-2 (PMC4865018; doi:10.1186/s12964-016-0133-2)
Supplement: Additional file 4: — Alignment of CNBP candidates with known CNB domains. (PDF 242 kb) [file 12964_2016_133_MOESM4_ESM.pdf]

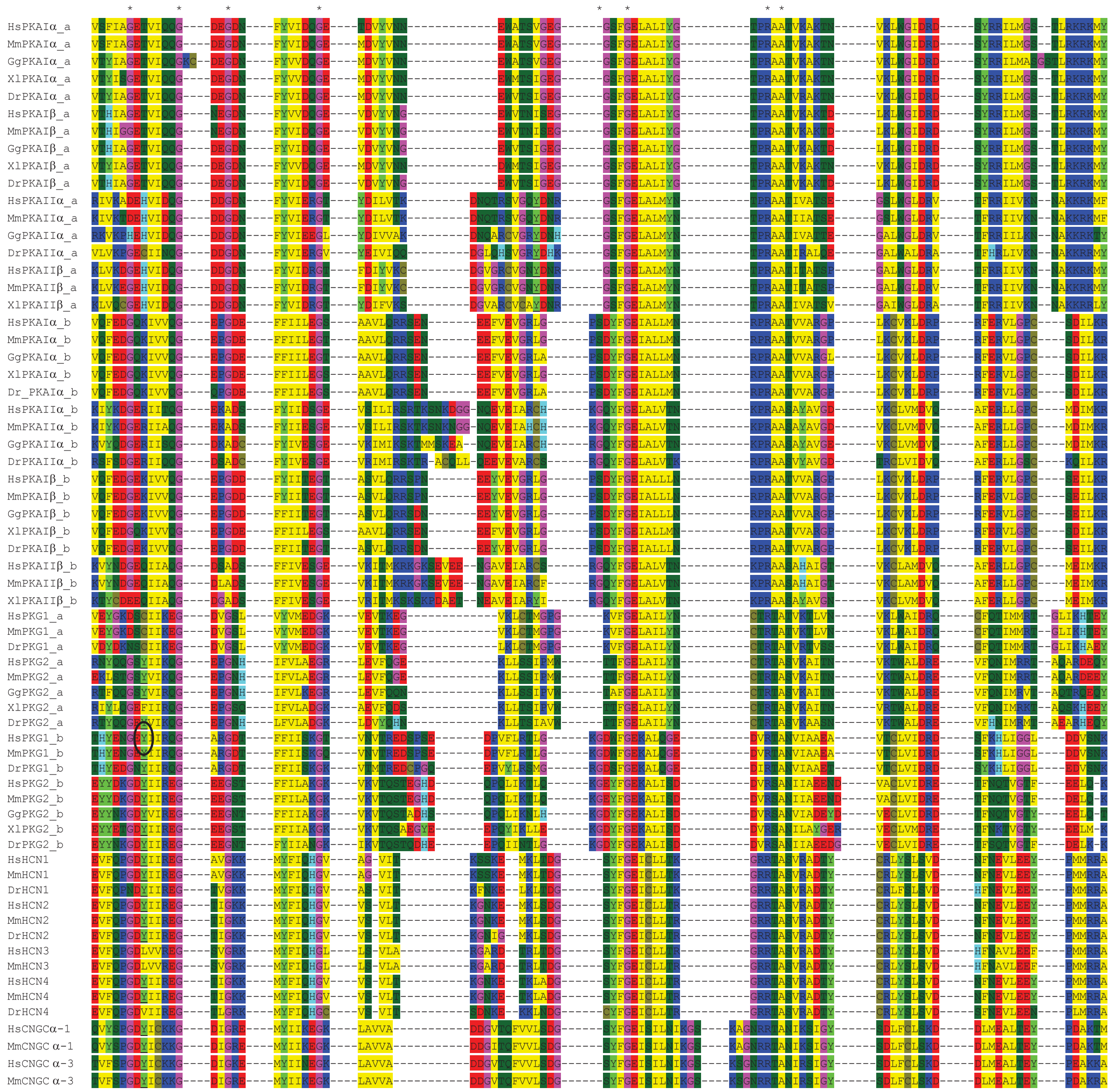
HsPKAIα_a
MmPKAIα_a
GgPKAIα_a
XlPKAIα_a
DrPKAIα_a
HsPKAIβ_a
MmPKAIβ_a
GgPKAIβ_a
XlPKAIβ_a
DrPKAIβ_a
HsPKAIIα_a
MmPKAIIα_a
GgPKAIIα_a
DrPKAIIα_a
HsPKAIIβ_a
MmPKAIIβ_a
XlPKAIIβ_a
HsPKAIα_b
MmPKAIα_b
GgPKAIα_b
XlPKAIα_b
Dr_PKAIα_b
HsPKAIIα_b
MmPKAIIα_b
GgPKAIIα_b
DrPKAIIα_b
HsPKAIβ_b
MmPKAIβ_b
GgPKAIβ_b
XlPKAIβ_b
DrPKAIβ_b
HsPKAIIβ_b
MmPKAIIβ_b
XlPKAIIβ_b
HsPKG1_a
MmPKG1_a
DrPKG1_a
HsPKG2_a
MmPKG2_a
GgPKG2_a
XlPKG2_a
DrPKG2_a
HsPKG1_b
MmPKG1_b
DrPKG1_b
HsPKG2_b
MmPKG2_b
GgPKG2_b
XlPKG2_b
DrPKG2_b
HsHCN1
MmHCN1
DrHCN1
HsHCN2
MmHCN2
DrHCN2
HsHCN3
MmHCN3
HsHCN4
MmHCN4
DrHCN4
HsCNGCα-1
MmCNGC α-1
HsCNGC α-3
MmCNGC α-3

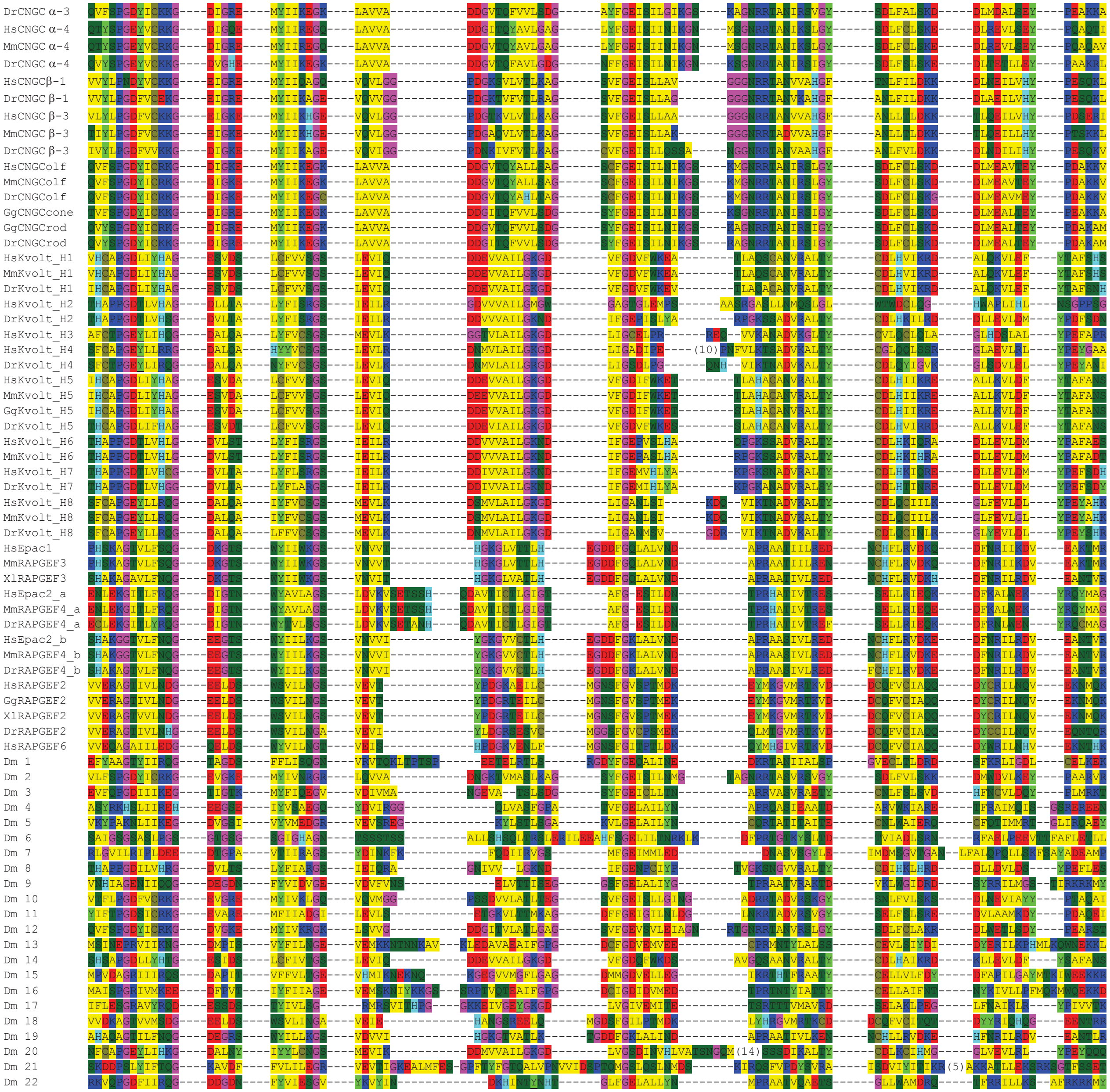
DrCNGC α-3
HsCNGC α-4
MmCNGC α-4
DrCNGC α-4
HsCNGCβ-1
DrCNGC β-1
HsCNGC β-3
MmCNGC β-3
DrCNGC β-3
HsCNGColf
MmCNGColf
DrCNGColf
GgCNGCcone
GgCNGCrod
DrCNGCrod
HsKvolt_H1
MmKvolt_H1
DrKvolt_H1
HsKvolt_H2
DrKvolt_H2
HsKvolt_H3
HsKvolt_H4
DrKvolt_H4
HsKvolt_H5
MmKvolt_H5
GgKvolt_H5
DrKvolt_H5
HsKvolt_H6
MmKvolt_H6
HsKvolt_H7
DrKvolt_H7
HsKvolt_H8
MmKvolt_H8
DrKvolt_H8
HsEpac1
MmRAPGEF3
XlRAPGEF3
HsEpac2_a
MmRAPGEF4_a
DrRAPGEF4_a
HsEpac2_b
MmRAPGEF4_b
DrRAPGEF4_b
HsRAPGEF2
GgRAPGEF2
XlRAPGEF2
DrRAPGEF2
HsRAPGEF6
Dm 1
Dm 2
Dm 3
Dm 4
Dm 5
Dm 6
Dm 7
Dm 8
Dm 9
Dm 10
Dm 11
Dm 12
Dm 13
Dm 14
Dm 15
Dm 16
Dm 17
Dm 18
Dm 19
Dm 20
Dm 21
Dm 22

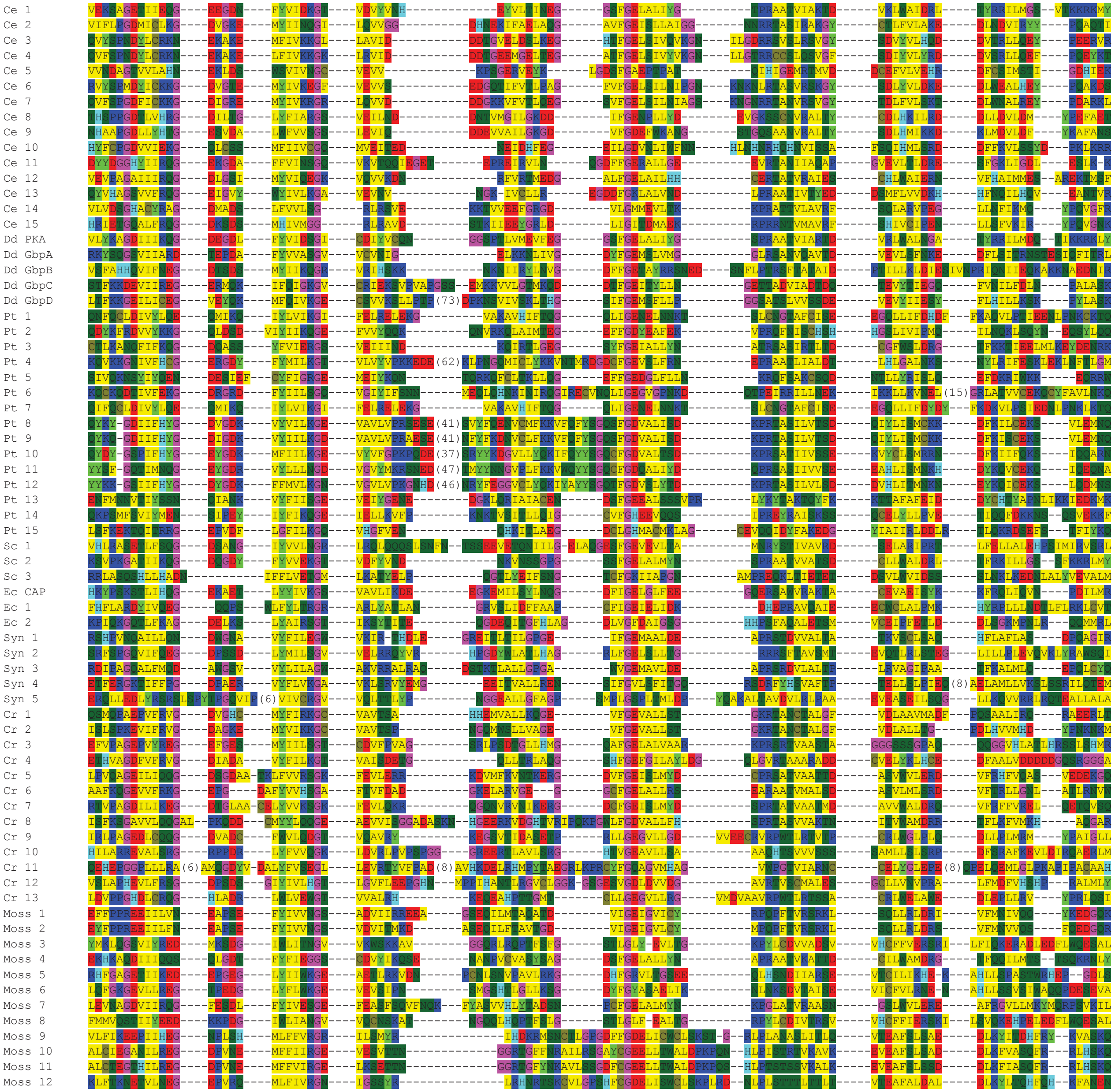
Ce 1
Ce 2
Ce 3
Ce 4
Ce 5
Ce 6
Ce 7
Ce 8
Ce 9
Ce 10
Ce 11
Ce 12
Ce 13
Ce 14
Ce 15
Dd PKA
Dd GbpA
Dd GbpB
Dd GbpC
Dd GbpD
Pt 1
Pt 2
Pt 3
Pt 4
Pt 5
Pt 6
Pt 7
Pt 8
Pt 9
Pt 10
Pt 11
Pt 12
Pt 13
Pt 14
Pt 15
Sc 1
Sc 2
Sc 3
Ec CAP
Ec 1
Ec 2
Syn 1
Syn 2
Syn 3
Syn 4
Syn 5
Cr 1
Cr 2
Cr 3
Cr 4
Cr 5
Cr 6
Cr 7
Cr 8
Cr 9
Cr 10
Cr 11
Cr 12
Cr 13
Moss 1
Moss 2
Moss 3
Moss 4
Moss 5
Moss 6
Moss 7
Moss 8
Moss 9
Moss 10
Moss 11
Moss 12

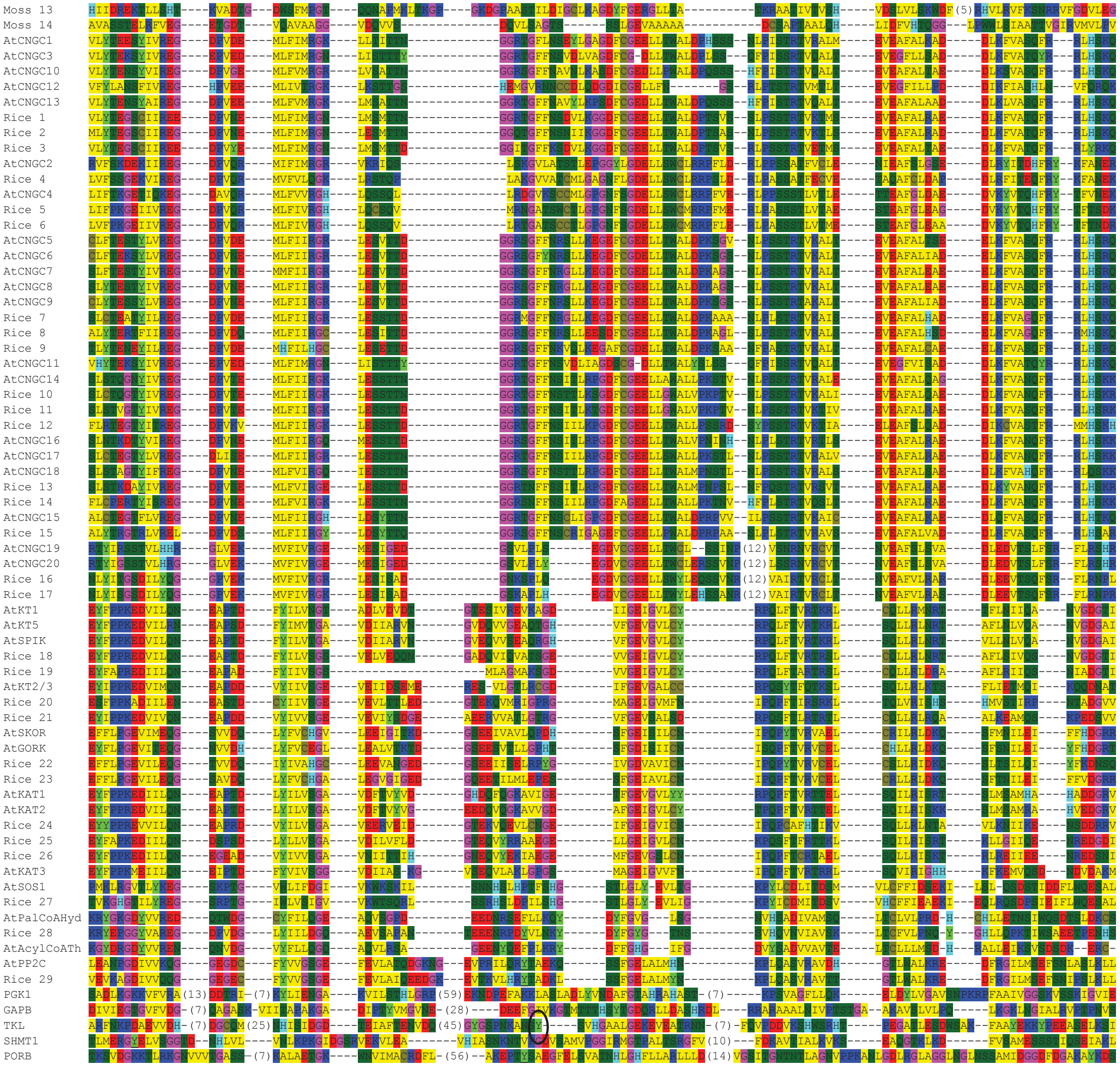
Moss 13
Moss 14
AtCNGC1
AtCNGC3
AtCNGC10
AtCNGC12
AtCNGC13
Rice 1
Rice 2
Rice 3
AtCNGC2
Rice 4
AtCNGC4
Rice 5
Rice 6
AtCNGC5
AtCNGC6
AtCNGC7
AtCNGC8
AtCNGC9
Rice 7
Rice 8
Rice 9
AtCNGC11
AtCNGC14
Rice 10
Rice 11
Rice 12
AtCNGC16
AtCNGC17
AtCNGC18
Rice 13
Rice 14
AtCNGC15
Rice 15
AtCNGC19
AtCNGC20
Rice 16
Rice 17
AtKT1
AtKT5
AtSPIK
Rice 18
Rice 19
AtKT2/3
Rice 20
Rice 21
AtSKOR
AtGORK
Rice 22
Rice 23
AtKAT1
AtKAT2
Rice 24
Rice 25
Rice 26
AtKAT3
AtSOS1
Rice 27
AtPalCoAHyd
Rice 28
AtAcylCoATh
AtPP2C
Rice 29
PGK1
GAPB
TKL
SHMT1
PORB

Additional file 4. Alignment of cyclic nucleotide binding protein candidates with known CNB domains

Representative CNB domains were downloaded from Interpro [IPR000595]. Where the protein has tandem CNB domains these are indicated by the letters “a” and “b”. The number in parenthesis indicates the number of amino acids that have been omitted. The * indicates conserved amino acids across species. The tyrosine residues that have been circled are sites of Y-nitration. Human – *H. sapiens* (Hs): PKA Iα [EMBL:P10644], PKA Iβ [EMBL:P31321], PKA IIα [EMBL:A8KAH7], PKA IIβ [EMBL:P31323], PKG I [EMBL:Q13976], PKG II [EMBL:Q13237], HCN 1 [EMBL:O60741], HCN 2 [EMBL:Q9UL51], HCN 3 [EMBL:Q9P1Z3], HCN 4 [EMBL:Q9Y3Q4], CNG α-1 [EMBL:P29973], CNG α-3 [EMBL:Q16281], CNG α-4 [EMBL:Q8IV77], CNG β-1 [EMBL:Q14028], CNG β-3 [EMBL:Q9NQW8], CNG olfactory (olf) [EMBL:Q16280], K^+ voltage gated channel (K volt) H1 [EMBL:O95259], K volt H2 [EMBL:Q708S9], K volt H3 [EMBL:Q9ULD8], K volt H4 [EMBL:Q9UQ05], K volt H5 [EMBL:Q8NCM2], K volt H6 [EMBL:Q9H252], K volt H7 [EMBL:Q9NS40], K volt H8 [EMBL:Q96L42], Epac1 [EMBL:O95398], Epac2 [EMBL:Q8WZA2], RAPGEF 2 [EMBL:Q9Y4G8] and RAPGEF 6 [EMBL:Q8TEU7]. Mouse - *Mus musculus* (Mm): PKA Iα [EMBL:Q9DBC7], PKA Iβ [EMBL:P12849], PKA IIα [EMBL:Q8K1M3], PKA IIβ [EMBL:P31324], PKG I [EMBL:P0C605], PKG II [EMBL:Q61410], HCN 1 [EMBL:O88704], HCN 2 [EMBL:O88703], HCN 3 [EMBL:O88705], HCN 4 [EMBL:B2RY58], CNG α-1 [EMBL:P29974], CNG α-3 [EMBL:Q9JJZ8], CNG α-4 [EMBL:Q3UW12], CNG β-3 [EMBL:Q9JJZ9], CNG olf [EMBL:Q62398], K volt H1 [EMBL:Q60603], K volt H5 [EMBL:Q920E3], K volt H6 [EMBL:Q32ME0], K volt H8 [EMBL:P59111], RAPGEF 3 [EMBL:Q8VCC8] and RAPGEF 4 [EMBL:Q9EQZ6]. Chicken - *Gallus gallus* (Gg): PKA Iα [EMBL:E1BRS5], PKA Iβ [EMBL:E1C2U6], PKA IIα [EMBL:E1C9H8], PKG II [EMBL:E1C4W9], CNG cone [EMBL:Q90805], CNG rod [EMBL:Q90980], K volt H5 [EMBL:E1BXK7] and RAPGEF 2 [EMBL:E1BVM2]. Frog - *Xenopus laevis* (Xl): PKA Iα [EMBL:Q6INK7], PKA Iβ [EMBL:Q6DJJ2], PKA IIβ [EMBL:Q6NTM8], PKG II [EMBL:Q6GPV8], RAPGEF 3 [EMBL:Q56TX1] and RAPGEF 2 [EMBL:Q6AX68]. Fish - *Danio rerio* (Dr): PKA Iα [EMBL:Q5I0F6], PKA Iβ [EMBL:Q08C49], PKA IIα [EMBL:Q6NW93], PKG I [EMBL:Q7T2E5], PKG II [EMBL:A2AVJ3], HCN 1 [EMBL:E7F8B3], HCN 2 [EMBL:F1QYP7], HCN 4 [EMBL:Q1L917], CNG α-3 [EMBL:E7F485], CNG α-4 [EMBL:F1Q5G4], CNG β-1 [EMBL:F1QK35], CNG β-3 [EMBL:E7F818], CNG olf [EMBL:Q0GFG2], CNG rod [EMBL:E7FE78], K volt H1 [EMBL:Q108P3], K volt H2 [EMBL:Q8JH78], K volt H4 [EMBL:E7FB36], K volt H5 [EMBL:Q5TYT4], K volt H7 [EMBL:E7F7K8], K volt H8 [EMBL:F1QTV6], RAPGEF 4 [EMBL:B0S4Y8] and RAPGEF 2 [EMBL:E9QI83]. Fly - *Drosophila melanogaster* (Dm): Dm 1 [EMBL:Q03042], Dm 2 [EMBL:Q9W201], Dm 3 [EMBL:A1Z9N7], Dm 4 [EMBL:Q9VL34], Dm 5 [EMBL:Q03043], Dm 6 [EMBL:Q9VJE6], Dm 7 [EMBL:Q2MGL9], Dm 8 [EMBL:Q7JPB9], Dm 9 [EMBL:P16905], Dm 10 [EMBL:Q9W2D5], Dm 11 [EMBL:Q9VXV8], Dm 12 [EMBL:Q24278], Dm 13 [EMBL:Q9VXJ8], Dm 14 [EMBL:Q02280], Dm15 [EMBL:Q9VGW3], Dm 16 [EMBL:Q8T4B9], Dm 17 [EMBL:E1JJF4], Dm 18 [EMBL:Q9VMF3], Dm 19 [EMBL:A1Z6P8], Dm 20 [EMBL:C7LA62], Dm 21 [EMBL:A8Y516] and Dm 22 [EMBL:P81900]. Worm - *Caenorhabditis elegans* (Ce): Ce 1 [EMBL:P30625], Ce 2 [EMBL:P90975], Ce 3 [EMBL:Q9N4C1], Ce 4 [EMBL:O61827], Ce 5 [EMBL:G5EDB9], Ce 6 [EMBL:A8WI01], Ce 7 [EMBL:Q03611], Ce 8 [EMBL:G5EE47], Ce 9 [EMBL:O44164], Ce 10 [EMBL:Q7Z205], Ce 11 [EMBL:O76360], Ce 12 [EMBL:H2KYN2], Ce 13 [EMBL:P34578], Ce 14 [EMBL:Q02331] and Ce 15 [EMBL:Q21534]. Slime mold - *Dictyostelium discoideum* (Dd): PKA [EMBL:P05987],

GbpA [EMBL:Q8MLZ3], GbpB [EMBL:Q8MM62], GbpC [EMBL:Q8MVR1] and GbpD [EMBL:Q54S40]. Protozoa - *Paramecium tetraurelia* (Pt): Pt 1 [EMBL:A0EIQ4], Pt 2 [EMBL:A0E8R0], Pt 3 [EMBL:Q3SEN1], Pt 4 [EMBL:A0CMH6], Pt 5 [EMBL:A0E7L3], Pt 6 [EMBL:A0BXC3], Pt 7 [EMBL:A0DN90], Pt 8 [EMBL:A0EB61], Pt 9 [EMBL:A0CP78], Pt 10 [EMBL:A0BEU4], Pt 11 [EMBL:A0BTC2], Pt 12 [EMBL:A0D1E5], Pt 13 [EMBL:A0BVR3], Pt 14 [EMBL:A0CW70] and Pt 15 [EMBL:A0CSD1]. Yeast - *Saccharomyces cerevisiae* (Sc): Sc 1 [EMBL:A6ZM06], Sc 2 [EMBL:A6ZVN6] and Sc 3 [EMBL:A6ZUD9]. Bacteria – *E. coli* (Ec): CAP [EMBL:P0ACJ8], Ec 1 [EMBL:P0A9E9] and Ec 2 [EMBL:P0A9E5]. Cyanobacteria – *Synechococcus* sp. JA-2-3B'a[2-13] (Syn): Syn 1 [EMBL:Q2JH72], Syn 2 [EMBL:Q2JKW7], Syn 3 [EMBL:Q2JLI3], Syn 4 [EMBL:Q2JIT2] and Syn 5 [EMBL:Q2JJK4]. Algae - *Chlamydomonas reinhardtii* (Cr): Cr 1 [EMBL:A8IYM3], Cr 2 [EMBL:A8IYN1], Cr 3 [EMBL:A8I6P0], Cr 4 [EMBL:A8HZS1], Cr 5 [EMBL:A8JDQ7], Cr 6 [EMBL:A8IXU5], Cr 7 [EMBL:A8J8T7], Cr 8 [EMBL:Q695H0], Cr 9 [EMBL:A8J0U9], Cr 10 [EMBL:A8JCV1], Cr 11 [EMBL:A8IH98], Cr 12 [EMBL:A8HX98] and Cr 13 [EMBL:A8J7I2]. Moss - *Physcomitrella patens*: moss 1 [EMBL:A5PH36], moss 2 [EMBL:A5PH37], moss 3 [EMBL:Q7XB48], moss 4 [EMBL:A9RTB2], moss 5 [EMBL:A9SD47], moss 6 [EMBL:A9SHM8], moss 7 [EMBL:A9SIB5], moss 8 [EMBL:D2U576], moss 9 [EMBL:A9T9L8], moss 10 [EMBL:A9T8G5], moss 11 [EMBL:A9SDJ9], moss 12 [EMBL:A9SNU5], moss 13 [EMBL:A9TTE5] and moss 14 [EMBL:A9TV43]. *A. thaliana* (At): CNGC1 [EMBL: O65717], CNGC2 [EMBL: O65718], CNGC3 [EMBL: Q9SKD7], CNGC4 [EMBL: Q94AS9], CNGC5 [EMBL: Q8RWS9], CNGC6 [EMBL: O82226], CNGC7 [EMBL: F4I2R9], CNGC8 [EMBL: Q9FXH6], CNGC9 [EMBL: Q9M0A4], CNGC10 [EMBL: Q9LNJ0-2], CNGC11 [EMBL: Q9SKD6], CNGC12 [EMBL: Q8GWD2], CNGC13 [EMBL: Q9LD40], CNGC14 [EMBL: Q9SJA4], CNGC15 [EMBL: Q9SL29], CNGC16 [EMBL: Q9SU64], CNGC17 [EMBL: Q8L7Z0], CNGC18 [EMBL: Q9LEQ3], CNGC19 [EMBL: Q9LDR2], CNGC20 [EMBL: Q9LD37], KT1 [EMBL:Q38998], KT2/3 [EMBL:Q38898], KT5 [EMBL:Q9SCX5], SPIK [EMBL:Q8GXE6], GORK [EMBL:Q94A76], SKOR [EMBL:Q9M8S6], KAT1 [EMBL:Q39128], KAT2 [EMBL:Q38849], KAT3 [EMBL:P92960], SOS1 [EMBL:Q9LKW9], PP2C [EMBL:Q9SL76], palmitoyl CoA hydrolase (Pal CoA Hyd) [EMBL:F4HU51] and acyl CoA thioesterase (Acyl CoA Th) [EMBL:Q5FYU1]. Rice - *Oryza sativa*: rice 1 [EMBL:B9FTK2], rice 2 [EMBL:B9F4U7], rice 3 [EMBL:B9FTK3], rice 4 [EMBL:Q9AUV9], rice 5 [EMBL:Q60EI8], rice 6 [EMBL:Q5ZAU3], rice 7 [EMBL:Q2QRA3], rice 8 [EMBL:Q10G24], rice 9 [EMBL:Q7X641], rice 10 [EMBL:B9F3N3], rice 11 [EMBL:B9FRX2], rice 12 [EMBL:Q6ZG24], rice 13 [EMBL:Q0IPW9], rice 14 [EMBL:Q653S0], rice 15 [EMBL:Q6K6P3], rice 16 [EMBL:A3B9H5], rice 17 [EMBL:B9F3H5], rice 18 [EMBL:Q0JKV1], rice 19 [EMBL:B9FVS3], rice 20 [EMBL:A2ZX97], rice 21 [EMBL:Q75HP9], rice 22 [EMBL:Q7XUW4], rice 23 [EMBL:Q653P0], rice 24 [EMBL:Q5QNI1], rice 25 [EMBL:Q5JM04], rice 26 [EMBL:Q6K3T2], rice 27 [EMBL:Q0ILJ6], rice 28 [EMBL:Q7XPS0] and rice 29 [EMBL:Q6K3D4].
