# Supplementary material for: The arabidopsis cyclic nucleotide interactome
Source: Cell Commun Signal. 2016 May 11;14:10. doi: 10.1186/s12964-016-0133-2 (PMC4865018; doi:10.1186/s12964-016-0133-2)
Supplement: Additional file 5: — Alignment of CNBP candidates with known GAF domains. (PDF 101 kb) [file 12964_2016_133_MOESM5_ESM.pdf]

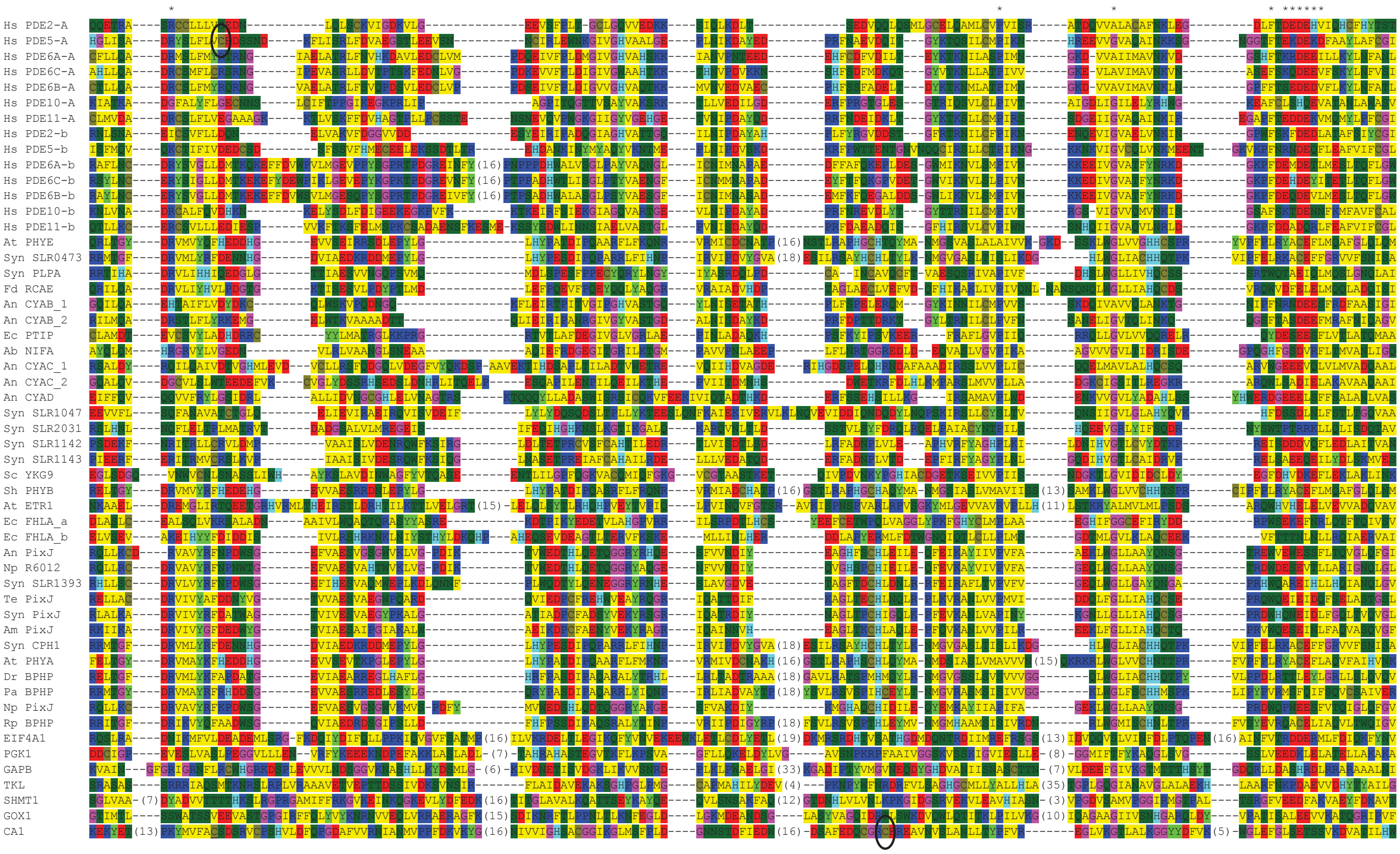

Additional file 5. Alignment of cyclic nucleotide binding protein candidates with known GAF domains

Representative GAF domains (IPR003018) were extracted from the literature. Where the protein has tandem GAF domains these are indicated by the letters “a” and “b”. The number in parenthesis indicates the number of amino acids that have been omitted. The * indicates conserved amino acids across species. The cysteine residues that have been circled are sites of S-nitrosylation. *H. sapiens* (Hs) phosphodiesterases PDE2, PDE5, PDE6A, PDE6B, PDE6C, PDE10 and PDE11 (Zoraghi et al., 2004); *A. thaliana* (At) phyE; *Synechocystis* sp. PCC 6803 (Syn) slr0473, plpA,

slr1047, slr2013, slr1142, slr1143; *Fremyella diplosiphon* (Fd) rcaE; *Anabaena* sp. PCC 7120 (An) adenylyl cyclases CYAB 1, CYAB 2, CYAC 1, CYAC 2, CYAD; *E. coli* PTIP and *Azospirillum brasilense* (Ab) NIFA (Aravind and Ponting, 1997); *S. cerevisiae* (Sc) YKG9; *Sorghum halepense* (Sh) phyB; At ETR1 and *E. coli* (Ec) Fhla (Ho et al., 2000); An PixJ; *Nostoc punctiforme* (Np) R6012; Syn slr1393; *Thermosynechococcus elongates* (Te) PixJ; Syn PixJ; *Acaryochloris marina* (Am) PixJ; Syn Cph1; At phyA; *Deinococcus radiodurans* (Dr) BphP and *Pseudomonas aeruginosa* (Pa) BphP (Narikawa et al., 2012) and Np PixJ and *Rhodopseudomonas palustris* (Rp) BphP (Narikawa et al., 2008).
